# Effects of chitin and chitosan on root growth, biochemical defense response and exudate proteome of *Cannabis sativa*

**DOI:** 10.1002/pei3.10106

**Published:** 2023-04-02

**Authors:** Pipob Suwanchaikasem, Shuai Nie, Alexander Idnurm, Jamie Selby‐Pham, Robert Walker, Berin A. Boughton

**Affiliations:** ^1^ School of BioSciences University of Melbourne Melbourne Victoria 3010 Australia; ^2^ Mass Spectrometry and Proteomics Facility, Bio21 Molecular Science and Biotechnology Institute University of Melbourne Melbourne Victoria 3052 Australia; ^3^ Cannabis and Biostimulants Research Group Pty Ltd Melbourne Victoria 3020 Australia; ^4^ Australian National Phenome Centre Murdoch University Perth Western Australia 6150 Australia

**Keywords:** Chitinase, elicitor, pathogenesis‐related, peroxidase, phytohormone, plant immunity, proteomics, Root‐TRAPR system

## Abstract

Fungal pathogens pose a major threat to *Cannabis sativa* production, requiring safe and effective management procedures to control disease. Chitin and chitosan are natural molecules that elicit plant defense responses. Investigation of their effects on *C. sativa* will advance understanding of plant responses towards elicitors and provide a potential pathway to enhance plant resistance against diseases. Plants were grown in the in vitro Root‐TRAPR system and treated with colloidal chitin and chitosan. Plant morphology was monitored, then plant tissues and exudates were collected for enzymatic activity assays, phytohormone quantification, qPCR analysis and proteomics profiling. Chitosan treatments showed increased total chitinase activity and expression of pathogenesis‐related (PR) genes by 3–5 times in the root tissues. In the exudates, total peroxidase and chitinase activities and levels of defense proteins such as PR protein 1 and endochitinase 2 were increased. Shoot development was unaffected, but root development was inhibited after chitosan exposure. In contrast, chitin treatments had no significant impact on any defense parameters, including enzymatic activities, hormone quantities, gene expression levels and root secreted proteins. These results indicate that colloidal chitosan, significantly enhancing defense responses in *C. sativa* root system, could be used as a potential elicitor, particularly in hydroponic scenarios to manage crop diseases.

## Graphical abstract

Chitosan elicits defense responses of *Cannabis sativa* root systems, but chitin has no effect in this regard. Defense enzymes and proteins were found to be increasingly produced in the tissues and secreted into exudate upon chitosan treatment. These results highlight the potential of chitosan to be alternatively used for managing fungal infection in agriculture, thereby minimizing the usage of chemicals.

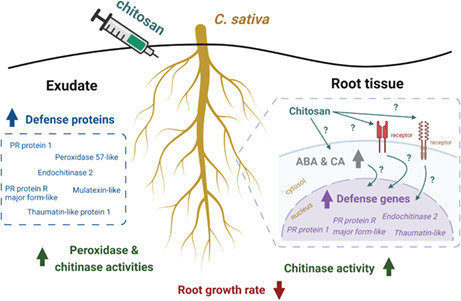

## 1 INTRODUCTION

*Cannabis sativa* L. has been widely grown for millennia, serving humankind with a range of benefits (Chandra, Lata, et al., 2017). There are two major *C. sativa* varieties, medicinal cannabis and industrial hemp, which are differentiated by the amount of tetrahydrocannabinol (THC) content in plant dry weight. Medicinal cannabis (high‐THC cannabis) is used for treating inflammation, seizure, nausea, vomiting and spasticity (Slawek et al., 2022). Industrial hemp (low‐THC cannabis) has a robust fiber, used for making cloth, textiles, rope, yarn, paper and building blocks (Zimniewska, 2022). Additionally, hemp seed is consumed as a food supplement due to high amount of proteins and beneficial polyunsaturated fatty acids (Callaway, 2004). Due to increasing market demand, cannabis agriculture has grown rapidly in the last decade, with increasing reports of negative impacts to plant cultivation, identified by local farmers and processors (Bodwitch et al., 2021; Jerushalmi et al., 2020). Pest and fungal attacks are one of the major problems for *C. sativa* production (Wang, 2021). Aphids, flea beetles, hemp borers, spider mites and bollworms are the common pests (McPartland et al., 2000) and fungal diseases such as gray mold, root rot, charcoal rot, stem canker and powdery mildew have been frequently recorded (Punja et al., 2019). Standard practices to control pests and pathogens have not been well established (Punja, 2021). Synthetic pesticides and fungicides like chlorpyrifos‐methyl and fluopyram or chemical agents like hydrogen peroxide (H_2_O_2_) and potassium bicarbonate have been applied in the field, posing a major concern for environmental and consumer safety (Craven et al., 2019; Sandler et al., 2019).

To avoid using chemicals, a number of natural products have been studied to induce plant defense, prompting plants to be more resistant against biotic stress (Thakur & Sohal, 2013; Yakhin et al., 2016). Chitin, chitosan, and their derivatives are one of the compound types that can elicit plant defense responses (Li et al., 2020). Chitin is an abundant natural polysaccharide, formed by a β‐1,4‐linkage of *N*‐acetyl‐D‐glucosamine (*N*‐GlcNAc) subunits. It is a main structural component of crustacean shells, insect exoskeletons and fungal cell walls (Younes & Rinaudo, 2015). Chitosan is a deacetylated form of chitin. It is less abundant in nature but can be processed from chitin using chemical reactions (Elieh‐Ali‐Komi & Hamblin, 2016). Several studies have demonstrated the beneficial effects of chitin and chitosan to promote plant defense. For example, treating tomato fruits with chitin suspensions enhanced the gene expressions and protein productions of superoxide dismutase, peroxidase, catalase and chitinase enzymes, reducing gray mold disease caused by *Botrytis cinerea* (Sun et al., 2018). Priming tomato leaves with chitosan solution stimulated callose deposition on plant cell wall and accumulation of defense hormones, leading to a reduction of leaf lesions caused by the same fungal pathogen (De Vega et al., 2021). However, most of those discoveries have been made in foliar parts of the plant due to ease of treatment and subsequent sampling. How these elicitors impact root systems is less well understood due to the physical challenges of studying roots and difficulty in investigating secretion of plant products into the surrounding soil (Lopez‐Moya et al., 2017). We recently developed an in vitro plant growth device, the Root‐TRAPR system, for imaging and sampling root material, making it possible to explore the impact of different environmental inputs on root system (Suwanchaikasem et al., 2022).

In this study, *C. sativa* was hydroponically grown in the Root‐TRAPR system and treated with colloidal chitin and chitosan. We hypothesized that these elicitors would, as for aboveground material, trigger the overall plant defense responses. Their effects on plant root system have been revealed in other crops; for example, mixing chitin into soil altered the microbial community surrounding the rhizosphere of lettuce (Debode et al., 2016) and supplying chitosan into plant growth medium induced release of phytohormones, lipid signaling and phenolic compounds into tomato root exudate (Suarez‐Fernandez et al., 2020). To advance understanding in this area, a proteomics approach was included in our study to characterize secreted proteins in the exudate. Expressions of defense‐related genes were quantified in root tissues using quantitative real‐time PCR (qPCR). In addition, root growth parameters, phytohormone levels and defense enzyme activities were also measured to examine plant responses inclusively.

## 2 MATERIALS AND METHODS

### 2.1 Chemicals

Chitin powder from shrimp shells (practical grade, product code: C7170) and chitosan (medium molecular weight, product code: 448877) were purchased from Sigma‐Aldrich, US. Acetonitrile (ACN), methanol, formic acid and trifluoroacetic acid (TFA) were liquid chromatography–mass spectrometry (LC–MS) grade solvents (Thermo Fisher Scientific, US). Ethanol was analytical grade (Chem‐Supply, Australia). Deionized water was used in the plant growth experiment. Milli‐Q water (Merck Millipore, Germany) was used for sample extraction and all instrumental analyses. Hoagland formulation was prepared as previously described (Suwanchaikasem et al., 2022).

### 2.2 Colloidal chitin and chitosan preparation

Approximately 5 g of chitin or chitosan powder was weighed in a 500‐mL Erlenmeyer flask and added with 50 mL of 85% phosphoric acid. Then, another 50 mL of 85% phosphoric acid was slowly added with continuous stirring. The mixture was incubated at 4°C overnight. Pre‐cooled 500 mL of ethanol was added to dilute the colloid and incubated at 4°C overnight again. The mixture was filtered through a double layer of Whatman No 1 filter paper using a Buchner funnel and then washed with water (approximately 3 L) until pH approached neutral (approximately 5–6). The retentate of colloidal chitin or chitosan was collected in a 50‐mL conical tube and frozen at −80°C overnight. Lyophilization was carried out using an Alpha 1‐4 LD plus freeze‐drier (Christ, Germany). Dried colloidal chitin and chitosan were kept at room temperature. Before use, chitin and chitosan were resuspended in a Hoagland solution in final concentrations of 0.1%, 0.2% and 0.5% w/v. The mixture was sonicated for 20 min to allow dispersion before applying to the plant.

### 2.3 Plant growth and harvest

*Cannabis sativa* cv. Ferimon seeds were kindly provided by Southern Hemp Australia. Seeds were surface sterilized using 70% ethanol and 0.04% sodium hypochlorite and germinated in a Petri dish for three days. Seedlings with tap root 4–6 cm in length were transferred to the Root‐TRAPR system and grown in a CMP 6010 growth chamber (Conviron, Canada). Growth condition was 16 h light at 25°C and 8 h dark at 21°C with constant 60% relative humidity. After maintenance for eight days in standard Hoagland solution, colloidal chitin and chitosan were added into the root growth chamber at 0.1%, 0.2% and 0.5% w/v concentrations. After eight days of the treatment, shoot and root tissues were harvested and ground using mortar and pestle with liquid nitrogen supply and subjected to phytohormones, enzymatic assays and qPCR analyses. Exudate was collected twice on treatment day before applying chitin and chitosan (pre‐exudate) and on the last day of experiment (post‐exudate) and kept at −80°C. Six plants were grown for each treatment.

### 2.4 Root growth measurement

Root morphology was imaged using the WinRHIZO Arabidopsis 2019 software (Regent Instruments, Canada). Root length and root surface area were calculated using the same protocol as previously described (Suwanchaikasem et al., 2022). Briefly, root region was manually assigned, and roots were automatically detected by the software based on image contrast where the root is brighter than the background. Manual adjustment was carried out when the software misplaced the root. The sum of all root lengths is the total root length, and the root length multiplied by the root diameter is the root surface area. Shoot and root fresh weight (FW) was measured using an analytical balance (Ohaus, US) upon sample collections.

### 2.5 Phytohormone analysis

Phytohormone contents were measured from shoot and root tissues using a targeted LC–MS/MS method with slight modification from the previous protocol (Suwanchaikasem et al., 2022). Approximately 100 mg tissue was weighed in a 1.5‐mL microcentrifuge tube and extracted with 400 μL of 70% methanol, supplied with 500 ng ml^−1^ of six internal standards ([^2^H]_5_‐zeatin, [^2^H]_2_‐indole‐3‐acetic acid, [^2^H]_7_‐cinnamic acid, [^2^H]_4_‐salicylic acid, [^2^H]_6_‐abscisic acid and dihydro‐jasmonic acid). The mixture was vigorously vortexed and centrifuged at 13,000× *g* for 20 min. Supernatant was collected in a LC–MS glass vial and subjected to the instrumental analysis, where the Triple‐Quad 6410 LC–MS machine (Agilent Technologies, US) was equipped with Poroshell 120 EC‐C18 column (2.7 μm; 2.1 × 100 mm). The column temperature was 45°C, and the injection volume was 5 μL. Mobile phases A and B were 0.1% FA in water and ACN, respectively. The flow rate was 300 μL min^−1^, and the LC gradient program was as follows: 80% A (0–2 min), 80%–50% A (2–3 min), 50%–5% A (3–12 min), 5% A (12–16 min), 5%–80% A (16–17 min) and 80% A (17–23 min). The gas temperature was 250°C with the flow of 13 L min^−1^. The nebulizer was set at 55 psi. The capillary voltage was 5500 and 4500 V for positive and negative ionization modes, respectively. Multiple reaction monitoring (MRM) transition, collision energy and polarity were set as follows: zeatin (220.1 → 136.1 *m/z*, 14 eV, positive), indole‐3‐acetic acid (IAA, 176.1 → 130.1 *m/z*, 10 eV, positive), cinnamic acid (CA, 149.1 → 103.1 *m/z*, 20 eV, positive), methyl‐IAA (190.1 → 130.0 *m/z*, 16 eV, positive), salicylic acid (SA, 137.0 → 93.0 *m/z*, 16 eV, negative), abscisic acid (ABA, 263.1 → 153.1 *m/z*, 8 eV, negative), jasmonic acid (JA, 209.1 → 59.0 *m/z*, 8 eV, negative), JA‐isoleucine (JA‐Ile, 322.1 → 129.9 *m/z*, 24 eV, negative), 12‐oxo‐phytodienoic acid (ODPA, 291.0 → 164.9 *m/z*, 20 eV, negative), [^2^H]_5_‐zeatin (225.2 → 137.1 *m/z*, 20 eV, positive), [^2^H]_2_‐IAA (178.1 → 132.0 *m/z*, 12 eV, positive), [^2^H]_7_‐CA (156.1 → 109.0 *m/z*, 22 eV, positive), [^2^H]_4_‐SA (141.0 → 97.1 *m/z*, 16 eV, negative), [^2^H]_6_‐ABA (269.1 → 159.1 *m/z*, 8 eV, negative) and dihydro‐JA (211.1 → 59.0 *m/z*, 12 eV, negative). Each sample was injected three times. The average relative peak area was compared against the standard curve, created from 4–6 dilutions of the standard (10–5000 ng mL^−1^). The calibration range was varied based on hormone levels, detected from the samples.

### 2.6 Peroxidase and chitinase activities

Peroxidase and chitinase activities were measured from tissues and exudates using the method previously described (Suwanchaikasem et al., 2022). Briefly, total proteins were extracted from tissue samples using 100 mM potassium phosphate buffer, pH 6.5. For peroxidase assay, protein extracts were treated with 0.025% H_2_O_2_ and 50 mM guaiacol in a 96‐wells microplate. The rate of absorbance change at 470 nm over 3 min was evaluated. For chitinase assay, the extracts were treated with 1% w/v colloidal chitin at 37°C for 2 h and centrifuged at 8000× *g* for 10 min to stop the reaction. Sodium borate buffer, pH 8.5 was mixed to adjust pH of the mixtures at 95°C for 5 min. Acidic dimethylaminobenzaldehyde (DMAB) reagent was added to colorize released *N*‐GlcNAc at 37°C for 20 min. Absorbance was measured at 585 nm using an Enspire Multilabel plate reader (Perkin Elmer, US) and evaluated against a *N*‐GlcNAc standard curve (50–2000 nM). Peroxidase and chitinase activities were normalized to FW for shoot and root tissues and root surface area (RSA) for root exudate samples.

### 2.7 Preparation of exudate proteins

Exudate solution (approximately 15 mL) was centrifuged at 2500× *g*, 4°C for 20 min to remove debris. Supernatant was transferred to an Amicon Ultra‐15 mL, 10 kDa molecular weight cutoff (MWCO) device (Merck Millipore, Germany) and centrifuged at 4000× *g*, 4°C for 40 min to concentrate exudate proteins. Retentate (approximately 200 μL) was collected in a 1.5‐mL microcentrifuge tube. Protein content was measured using Bradford assay. Briefly, 20 μL of protein extract was mixed with 180 μL of five‐time diluted Bradford reagent (Bio‐Rad, US) in a 96‐well microplate. The mixture was incubated at room temperature for 10 min. Absorbance was detected at 595 nm using the plate reader. A bovine serum albumin (BSA) standard curve was created from 0 to 100 μg mL^−1^.

Eight μg of proteins were aliquoted from each sample and dried down using a RVC 2‐33 vacuum concentrator (Martin Christ, Germany). The proteins were processed through S‐Trap micro spin columns (Protifi, US) using manufacturer's protocol with slight modification. Forty‐six μL of 1× lysis buffer (5% sodium dodecyl sulfate in 50 mM triethylammonium bicarbonate (TEAB) buffer, pH 8.5) was added to resuspend the dried pellet and vigorously vortexed. Two μL of 240 mM Tris(2‐carboxyethyl)‐phosphine (TCEP) was added and incubated at 55°C for 15 min to reduce proteins. Then, 4 μL of 500 mM iodoacetamide was added and incubated at 37°C for 30 min in the dark to alkylate proteins. Six μL of 27.5% phosphoric acid was added to denature proteins, followed by adding 330 μL of binding buffer (100 mM TEAB in 90% methanol). S‐Trap micro spin column (Protifi, US) was placed into a 2‐ml microcentrifuge tube. The sample was applied to the column and centrifuged at 4000× *g* for 30 s to trap proteins. The sample was loaded twice (200 μL each time). Washing step was carried out three times by adding 150 μL of binding buffer into the column and centrifuged at 4000× *g* for 1 min. The column was centrifuged at 4000× *g* for 2 min and transferred into a new 1.5‐ml microcentrifuge tube. Fifty μL of 20 μg mL^−1^ trypsin in 50 mM TEAB buffer was carefully added into the column by avoiding trapping air bubble on top of the membrane. The column was loosely capped to limit evaporation loss and incubated at 37°C for 18 h to digest proteins. The column was rehydrated with 50 μL of 50 mM TEAB buffer for 30 min at room temperature and centrifuged at 4000× *g* for 1 min. Peptides were then eluted from the column using three elution buffers, 40 μL of 50 mM TEAB buffer, 0.2% FA and 50% ACN, respectively. Centrifugation was performed each time at 4000× *g* for 1 min. Three hundred μL of water was added to dilute the peptides. To clean up final peptides, the solution was loaded into an Amicon Ultra‐0.5 mL, 30 kDa MWCO device (Merck Millipore, Germany) and centrifuged at 13,000× *g* for 20 min. Filtrate was collected and dried down using the vacuum concentrator. Finally, pellet was resuspended with 40 μL of MS loading buffer (2% ACN in water + 0.05% TFA) and centrifuged at 13,000× *g* for 20 min. Fifteen μL peptide solution was transferred to a LC–MS vial and subjected to proteomics LC–MS/MS analysis.

### 2.8 Proteomics LC–MS/MS data acquisition

The Nano‐electrospray ionization (ESI)‐LC–MS/MS system, comprising of Ultimate 3000 RSLC (Thermo Fisher Scientific, US), Acclaim Pepmap RSLC analytical column (C18, 100 Å, 75 μm × 50 cm), Acclaim Pepmap nano‐trap column (C18, 100 Å, 75 μm × 2 cm) and Q Exactive Plus Orbitrap mass spectrometer (Thermo Fisher Scientific, US) was used for the proteomics LC–MS/MS analysis. Column temperature was 50°C. Mobile phase A and B was 0.1% FA + 5% dimethyl sulfoxide (DMSO) in water and ACN, respectively. Injection volume was 6 μL. The trap column was loaded with peptide sample at an isocratic flow of 2% ACN containing 0.05% TFA at 6 μL min^−1^ for 5 min, followed by the switch of the trap column as parallel to the analytical column. LC flow rate was 300 nL min^−1^ and gradient was set as follows: 97% A (0–1 min), 97%–77% A (1–30 min), 77%–60% A (30–40 min), 60%–20% A (40–45 min), 20% A (45–50 min), 20%–97% A (50–50.1 min) and 97% (50.1–60 min). MS ionization was in positive mode with the settings of 1.9 kV nano‐ESI voltage, 70% S‐lens radio frequency (RF) and capillary temperature of 250°C. Full scan MS spectra was set at mass range of 375–1400 *m/z*, maximum ion trapping time of 50 ms, autogain control target value of 3 × 10^6^ and resolution of 70,000 at *m/z* 200. Data dependent acquisition (DDA) mode was used to acquire higher‐energy collisional dissociation (HCD)‐MS/MS spectra of the top 15 most abundant precursor ions from each full scan MS spectrum. The *m/z* isolation window of 1.2, autogain control target value of 5 × 10^4^, 30% normalized collision energy, mass range of 200–2000 *m/z*, maximum ion trapping time of 50 ms, and resolution of 17,500 at *m/z* 200 were used to perform HCD‐MS/MS of precursor ions (charge states from 2 to 5). Dynamic exclusion of 30 s was enabled.

### 2.9 Proteomics data analysis

Raw LC–MS/MS data were processed using the MaxQuant version 2.0 software (Tyanova, Temu, & Cox, 2016). Protein database was gathered from the NCBI web service, including all *C. sativa* proteins annotated from *C. sativa* draft genome sequence (van Bakel et al., 2011). When searching against the database, methionine oxidation and protein N‐terminal acetylation were set as variable modifications and cysteine carbamidomethylation was a fixed modification. Trypsin was selected as the digestion method, allowing maximum 2 mis‐cleavages. Label‐free quantification (LFQ) was applied. Reversed hits, potential contaminants and proteins identified only by site were removed from the identification list. Proteins were rigidly filtered using criteria of unique and razor peptides ≥2 and peptide sequence coverage ≥8%. Proteins with null LFQ intensity in all samples were discarded. In total, 57 high‐confidence protein groups were identified from the dataset and protein identification details are presented in Table S1.

To understand characteristics of the identified proteins, online DeepGoWeb version 1.0.3 web portal (Kulmanov & Hoehndorf, 2020) was used to predict their cellular location, molecular function and biological process. Prediction threshold was set at zero. Prediction was based on sequence similarity to the annotated proteins in the UniProt database. The score, ranging from zero to one, indicated the possibility of the protein to associate with particular cellular location, molecular function and biological process.

### 2.10 Quantitative real‐time PCR

Based on proteomics results, four genes related to pathogenesis‐related (PR) protein 1, endochitinase 2, PR protein R major form‐like and thaumatin‐like protein 1 were selected for validation of gene expression level in the root tissues. Gene and primers details are supplied in Table S2. RNA was extracted from root tissues using a RNeasy Plant Mini kit (Qiagen, Germany) according to the manufacturer's protocol. RNA was eluted from the spin column using 40 μL of nuclease‐free water and RNA concentration was measured using a UV5Nano spectrophotometer (Mettler Toledo, US). A 500 ng RNA was converted to complementary DNA (cDNA) using Superscript II reverse transcriptase (Thermo Fisher Scientific, US) and kept at −20°C. Before undertaking PCR reaction, cDNA was diluted three times in water. Primer efficiency was determined on a test sample across five concentrations of 10‐fold dilutions. PCR primers with efficiency of 90%–110% and standard curve linearity (*r* ^2^) above 0.98 were used in the analysis. A 10 μL reaction was composed of 1× SYBR Green Supermix (Bio‐Rad, US), 0.4 μM forward and reverse primers and 1 μL of cDNA. The amplification was performed using CFX384 Touch Real‐Time PCR system (Bio‐Rad, US) with initial denaturation of 30 s at 95°C, followed by 40 cycles of 15 s at 95°C and 40 s at 60°C. Melt curve was performed with 0.5°C increment from 65 to 95°C. Lid temperature was set at 95°C. Quantitative cycle (Cq) and melt temperature data were analyzed using CFX Manager software version 3.1 (Bio‐Rad, US). Relative quantification was calculated according to (Livak & Schmittgen, 2001). *C. sativa* cucumber peeling cupredoxin (XP_030477701.1) was used as a reference gene as it was found across post‐exudate samples (Table S1). Five replicates were performed per treatment.

### 2.11 Statistical analysis

One‐way ANOVA, followed by Tukey's post hoc analysis was used to test significant difference (*p* < .05) across all treatments. Paired *t*‐test was used to test the difference between pre‐ and post‐exudates of each treatment (*p* < .05). Linear regression was built within the growth curves of root length and surface area. The regression line of each treatment was individually compared against that of control to indicate growth rate variation. Regression coefficient calculated from the interaction between day and treatment with *p* < .05 indicated significant difference of the growth rate between control and particular treatment. Statistical analysis and graph plotting were performed using Minitab Statistical software version 20.3 (Minitab, US).

For proteomics data, statistical analysis was performed on raw LFQ intensity using Perseus version 2.0 software (Tyanova, Temu, Sinitcyn, et al., 2016). For pre‐ and post‐exudates, one‐way ANOVA with permutation‐based false discovery rate (FDR) followed by Tukey's post hoc test was used for testing significant difference (*q*‐value <.05) across all treatments at each timepoint. Paired *t*‐test was used to compare difference between pre‐ and post‐exudate of each treatment. Online MetaboAnalyst version 5.0 software (Pang et al., 2021) was employed to construct principal component analysis (PCA) and hierarchical clustering heatmap. To achieve normal distribution, the data were square root (SQRT) transformed and mean centered for the PCA plot, showing the first and second components in *x* and *y* axes, respectively. LFQ intensity of post‐exudate samples were log transformed and subjected for hierarchical clustering heatmap analysis using Euclidean distance measure and Ward clustering method.

## 3 RESULTS

### 3.1 Chitosan affects *C. sativa* root development

**FIGURE 1:**
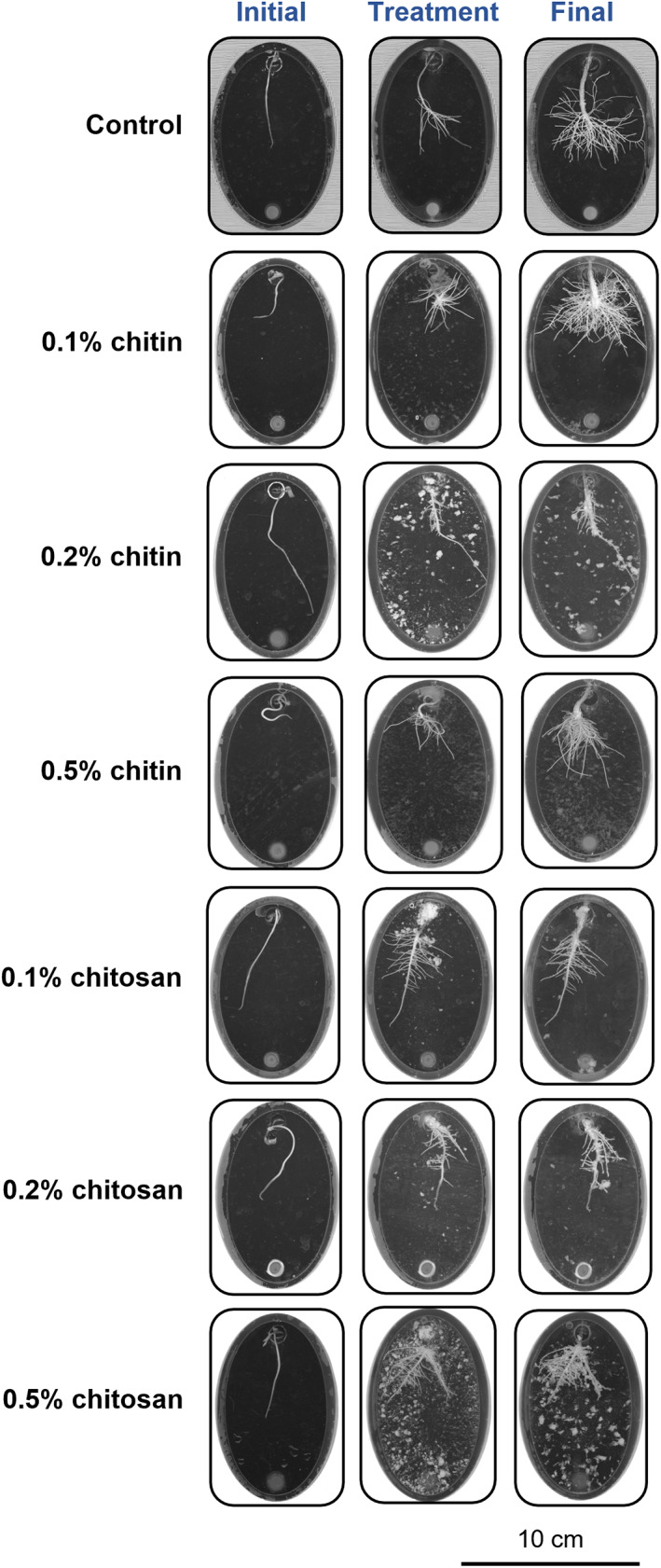
Scanned root images showing root development in the Root‐TRAPR system of control, chitin and chitosan treatments. Three scanning times were *initial day* when the seedling was transferred into the Root‐TRAPR system, *treatment day (day 0)* when chitin or chitosan was applied into the system and *final day (day 8)* when plant tissues were collected. Insoluble parts of colloidal chitin and chitosan might be spotted as white specks in the root chamber of the Root‐TRAPR system. The images were captured using a calibrated scanner, equipped with the WinRHIZO Arabidopsis 2019 software.

Chitin and chitosan are water‐insoluble polymers (Rinaudo, 2006); therefore, they were prepared in a colloidal form to create a colloidal suspension with the hydroponic solution. As observed, the viscosity of the mixture increased relative to the increase of chitin and chitosan concentrations. The higher chitin and chitosan concentrations, the more viscous colloidal suspensions. However, the mixture still contained insoluble particles, which could be seen as white specks in the root chamber of the Root‐TRAPR system (Figure 1) and were also observed in other studies upon direct dissolution (Chandrasekharan et al., 2019; Jaworska & Górak, 2018). Hence, the amount of soluble chitin and chitosan in the suspension might be slightly lesser than the concentration stated but was comparatively increased corresponding to the increased chitin and chitosan concentrations.

**FIGURE 2:**
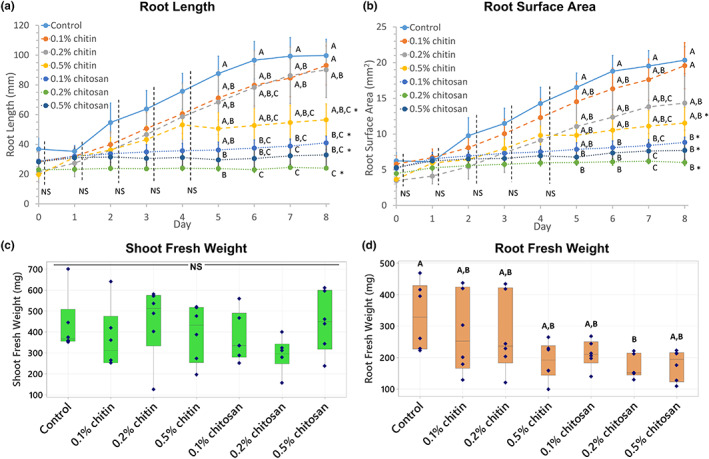
Plant growth measurements showing root length (a), root surface area (b), shoot fresh weight (c) and root fresh weight (d) of control, chitin and chitosan treatments, analyzed from six biological replicates. Chitin and chitosan were applied on day 0 and tissue samples were collected and measured for fresh weight on day 8. Growth curves (a and b) display values of mean ± standard error (SE) bar. Box plots (c and d) display interquartile range box with whiskers and six individual values. Letters (A–C) refer to statistically significant difference (*p* < .05) using one‐way ANOVA, followed by Tukey's post hoc analysis. Asterisk (*) indicates significant difference (*p* < .05) in growth rate, comparing respective treatment with control (Figure S1). NS represents non‐significant differences tested across all sample groups.

Changes in root development according to chitin and chitosan treatments were clearly visible from the scanned root images (Figure 1). In control plants, root developed from 36.83 ± 7.34 to 99.71 ± 10.96 mm in length and 6.24 ± 1.15 to 20.34 ± 1.90 cm^2^ in surface area within eight days after treatment (Figure 2a,b; Table S3). In chitin treatments, root growth was not significantly impacted by the lower chitin concentrations (0.1% and 0.2%) but affected by 0.5% concentration, where root growth rate of 0.5% chitin treatment (4.30 mm day^−1^) was significantly slower (*p* = .007) than control (9.10 mm day^−1^) (Figure S1). The final root length and surface area of 0.5% chitin treatment were 56.71 ± 10.64% (*p* = .125) and 56.65 ± 10.87% (*p* = .137) of the control, respectively (Figure 2a,b). By contrast, chitosan largely affected root development. Final root lengths of 0.1%, 0.2% and 0.5% chitosan treatments were significantly shorter than control, which were only 41.12 ± 4.55% (*p* = .013), 28.49 ± 1.30% (*p* = .001) and 31.42 ± 6.78% (*p* = .011) of the control, respectively (Figure 2a). Likewise, root surface area of 0.1%, 0.2% and 0.5% chitosan treatments were only 43.37 ± 4.36% (*p* = .021), 30.60 ± 2.20% (*p* = .004) and 38.91 ± 6.16% (*p* = .025) of the control, respectively (Figure 2b). Root barely expanded upon chitosan exposures with growth rate less than 1.5 mm day^−1^ (*p* < .001) (Figure S1).

Interestingly, shoot biomass was not impacted by any chitin and chitosan treatments (ANOVA *p* = .419), where shoot fresh weights were in the range of 291.52 to 450.97 mg among all treatments (Figure 2c). However, root biomass was significantly reduced, where root fresh weight of 0.2% chitosan treatment (168.68 ± 15.42 mg) was significantly lower (*p* = .039) than control (331.93 ± 43.82 mg). It was approximately 1.5–2 times lower than control in 0.5% chitin (188.97 ± 24.99 mg, *p* = .097), 0.1% chitosan (211.07 ± 17.99 mg, *p* = .231) and 0.5% chitosan (176.60 ± 19.76 mg, *p* = .056) treatments (Figure 2d).

This morphological data demonstrates that chitosan strongly inhibited root growth and reduced root biomass, but chitin had no significant impact in this regard.

### 3.2 Shoot auxin and root ABA and CA levels are changed in response to chitosan treatments

**FIGURE 3:**
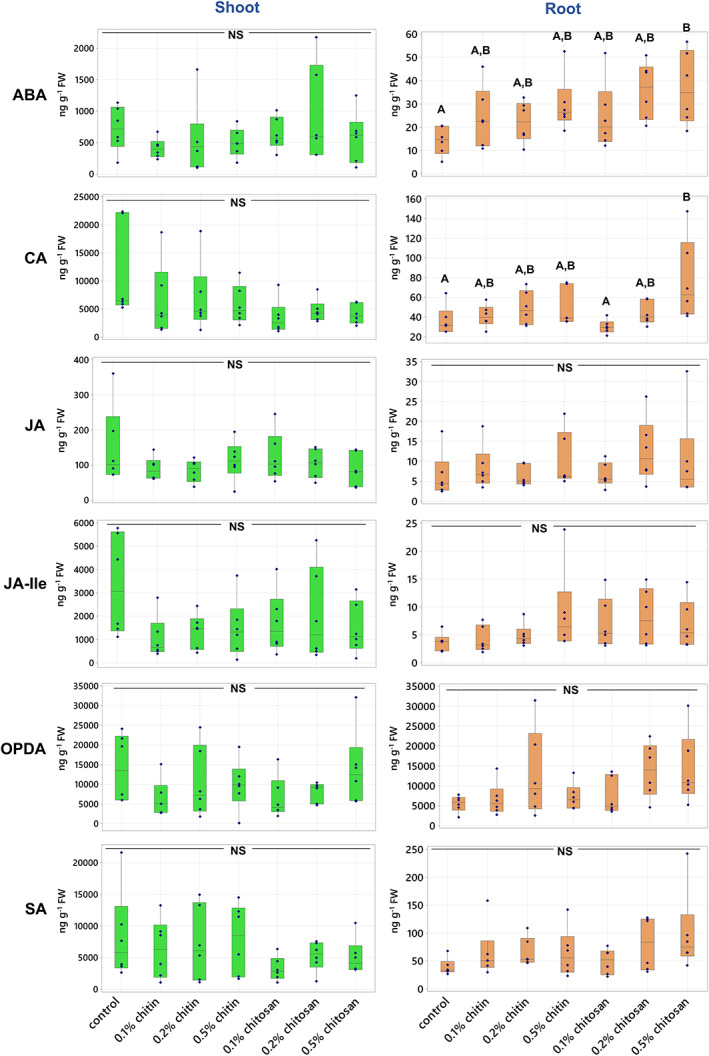
Phytohormone levels of abscisic acid (ABA), cinnamic acid (CA), jasmonic acid (JA), JA‐isoleucine (JA‐Ile), 12‐oxo‐phytodienoic acid (OPDA) and salicylic acid (SA) measured from shoot and root tissues of control, chitin and chitosan treatments within six biological replicates. Letters (A–B) refer to statistically significant difference (*p* < .05) using one‐way ANOVA, followed by Tukey's post hoc analysis. NS represents non‐significant differences tested across all sample groups.

Phytohormones are key messengers, driving signaling processes behind plant defense responses (Shigenaga & Argueso, 2016). To evaluate the effects of chitin and chitosan on *C. sativa* defense, the levels of defense hormones including ABA, SA, JA, JA‐Ile and OPDA and a plant growth regulator, CA were measured from shoot and root tissues, and the results are shown in Figure 3. The levels of growth hormones including IAA, methyl‐IAA and zeatin are presented in Figure S2. Raw phytohormone data is supplied in Table S3. In shoot tissues, the levels of almost all phytohormones measured were relatively comparable across the treatments. Only methyl‐IAA hormone showed significant difference, where its levels in 0.1% and 0.5% chitosan treatments (94.95 ± 25.44 and 117.33 ± 24.33 ng g^−1^ FW, respectively) were significantly lower (*p* = .022 and .046, respectively) than that of control (353.44 ± 45.20 ng g^−1^ FW). In addition, the level of IAA also exhibited a similar tendency to Me‐IAA, where the levels of IAA in 0.1% and 0.5% chitosan treatments (324.84 ± 46.10 and 280.47 ± 24.92 ng g^−1^ FW, with *p* = .935 and .653, respectively) were slightly decreased, with approximately 20%–30% lower than control (405.45 ± 70.97 ng g^−1^ FW) (Figure S2; Table S3). These data indicate that root chitosan treatment might affect shoot auxin levels.

In root tissues, ABA and CA levels in 0.5% chitosan treatment (ABA; 36.83 ± 6.41 and CA; 77.05 ± 16.94 ng g^−1^ FW) were significantly higher (*p* = .040 and .022, respectively) than those of control (ABA; 14.18 ± 2.48 and CA; 36.29 ± 6.04 ng g^−1^ FW) (Figure 3). Although significant differences were not detected from the other hormones, increasing tendencies were observed from SA, JA‐Ile and OPDA in chitosan treatments. The levels of SA in 0.2% and 0.5% chitosan treatments (80.97 ± 19.63 and 99.17 ± 29.67 ng g^−1^ FW, with *p* = .636 and .226, respectively) were approximately 2 times higher than control (39.19 ± 6.10 ng g^−1^ FW). Likewise, the OPDA levels of 0.2% and 0.5% chitosan treatments (13.87 ± 2.79 and 14.13 ± 3.67 μg g^−1^ FW, with *p* = .318 and .284, respectively) were nearly 3 times higher than control (5.52 ± 0.83 μg g^−1^ FW). The levels of JA‐Ile in 0.1%, 0.2% and 0.5% chitosan treatments (7.04 ± 1.72, 8.22 ± 1.88 and 6.88 ± 1.63 ng g^−1^ FW, with *p* = .841, .572 and .868, respectively) were 1.9–2.2 times higher than control (3.69 ± 0.60 ng g^−1^ FW). These data indicate that defense hormones likely increased in response to chitosan treatment locally at the roots, where the treatment was applied.

### 3.3 Total peroxidase and chitinase activities are increased in root tissues and exudates upon chitosan treatments

**FIGURE 4:**
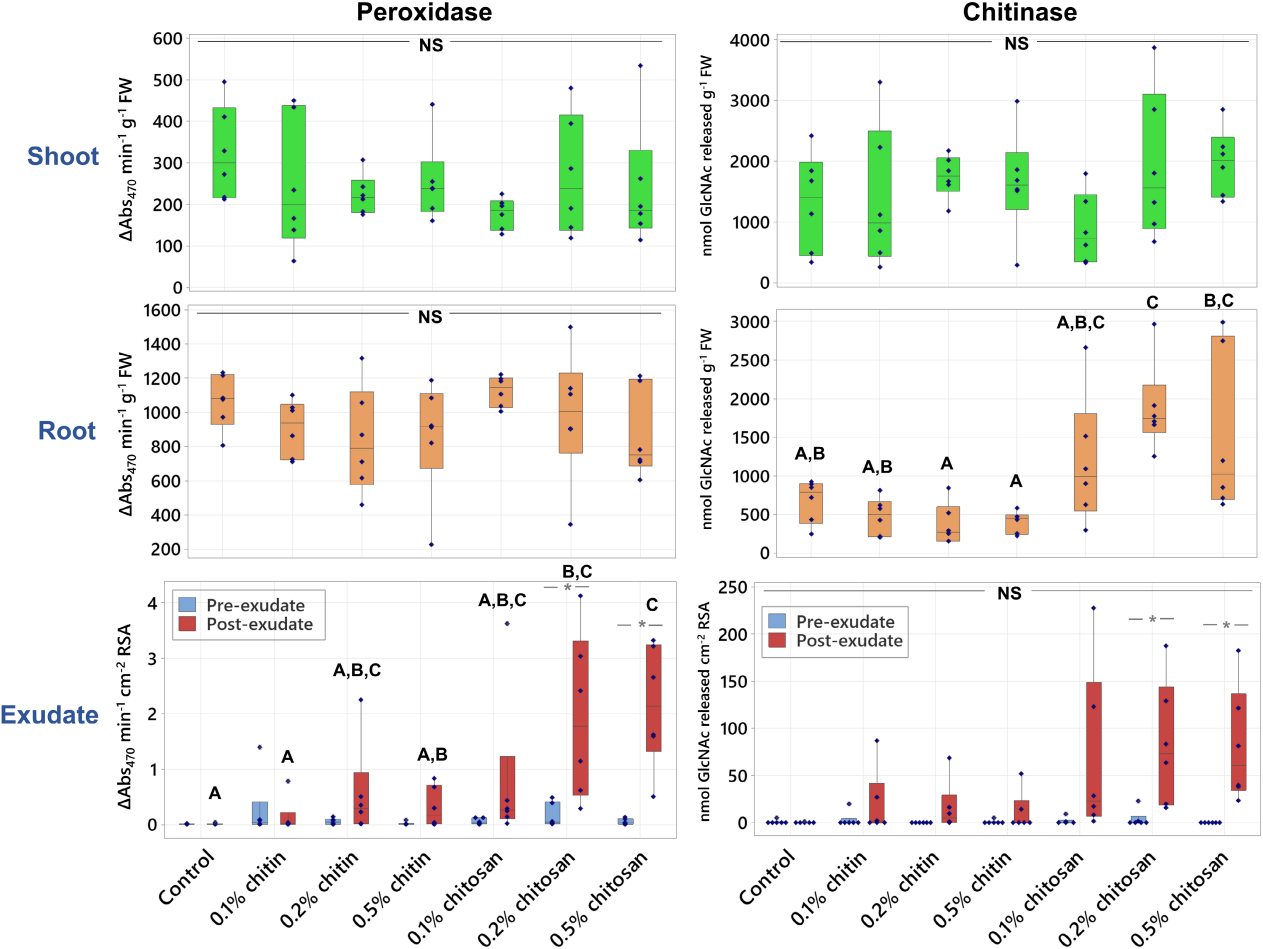
Peroxidase and chitinase activities measured from shoot and root tissues and pre‐ and post‐exudates of control, chitin and chitosan treatments within six biological replicates. Letters (A–C) refer to statistically significant difference (*p* < .05) using one‐way ANOVA, followed by Tukey's post hoc analysis. Asterisk (*) indicates statistically significant difference (*p* < .05) between pre‐ and post‐exudate of each treatment using paired *t*‐test. NS represents ANOVA non‐significant differences tested across all sample groups.

Protective function of defense enzymes is one of the mechanisms that plants use to manage biotic stresses (Appu et al., 2021). In this study, total activities of two defense enzymes, peroxidase and chitinase, were measured in shoot, root tissues and exudates. Bioassays to test peroxidase and chitinase activities in plant samples are well established and widely used (Senthilkumar et al., 2021). In shoot tissues, peroxidase and chitinase activities were comparable among all treatments (ANOVA *p* = .543 and .304, respectively) (Figure 4; Table S3). In root tissues, chitinase activity of 0.2% chitosan treatment (1880.29 ± 235.16 nmol GlcNAc released g^−1^ FW) was significantly higher (*p* = .017) than control (678.29 ± 113.42 nmol GlcNAc released g^−1^ FW). The activities in 0.1% and 0.5% chitosan treatments (1183.04 ± 340.63 and 1523.18 ± 433.89 nmol GlcNAc released g^−1^ FW, respectively) were also increased, but to a lower extent and not significantly different from the control (*p* = .744 and .188, respectively). However, peroxidase activities were comparable across all treatments (837.85 ± 127.48 to 1124.55 ± 36.85 ΔAbs_470_ min^−1^ g^−1^ FW, with ANOVA *p* = .401).

In exudates, peroxidase and chitinase activities were tested before and after the treatments and normalized to RSA instead of FW. This is because root secretion would mainly occur at root tip and elongation zone, found at the end of individual roots (Canarini et al., 2019). Therefore, root area would be a better indicator than root weight, dominated by taproot, to reflect the number of proteins secreted into exudate. Prior to treatment (pre‐exudate), peroxidase and chitinase activities were nearly undetectable in all treatments (Figure 4). After treatment (post‐exudate), peroxidase activity of 0.2% and 0.5% chitosan treatments (1.94 ± 0.67 and 2.15 ± 0.49 Δabs_470_ min^−1^ cm^−2^ RSA, respectively) was significantly higher than control (0.012 ± 0.006 Δabs_470_ min^−1^ cm^−2^ RSA), with *p* = .020 and .007, respectively and their pre‐exudates (0.167 ± 0.095 and 0.052 ± 0.023 Δabs_470_ min^−1^ cm^−2^ RSA, with *p* = .037 and .005, respectively). Peroxidase activity was slightly increased in chitin treatments. For example, the activity measured from 0.2% chitin treatment was 0.56 ± 0.38 Δabs_470_ min^−1^ cm^−2^ RSA, which was higher than control and its pre‐exudate (0.056 ± 0.024 Δabs_470_ min^−1^ cm^−2^ RSA). However, the increase was not significantly different in terms of statistics (*p* = .952), and the activity was still approximately 3–4 times lower than that of 0.2%–0.5% chitosan treatments (Figure 4; Table S3).

Total chitinase activities in the exudates of all chitosan treatments were substantially higher than that of control. They were 67.72 ± 40.25, 83.26 ± 29.56 and 81.02 ± 27.41 nmol GlcNAc released cm^−2^ RSA in the 0.1%, 0.2% and 0.5% chitosan treatments, respectively. It was only 0.208 ± 0.208 nmol GlcNAc released cm^−2^ RSA in control. However, the activity was low in some replicates of chitosan exudate samples, creating high intra‐variation within the sample groups and resulting in statistically insignificant difference with *p* = .286, .106 and .124, respectively (Figure 4; Table S3). Nonetheless, when comparing between before and after treatment, chitinase activities were significantly increased in 0.2% and 0.5% chitosan treatments, with *p* = .042 and .023, respectively (Figure 4). They were 1.79 ± 1.47 and 0.00 ± 0.00 GlcNAc released cm^−2^ RSA in pre‐exudates of 0.2% and 0.5% chitosan treatments, respectively and increased to 83.26 ± 29.56 and 81.02 ± 27.41 GlcNAc released cm^−2^ RSA after the treatments. The increasing tendency of chitinase activity was also detected from chitin treatments but not significantly different. For example, total chitinase activity of 0.2% chitin treatment was undetectable in the pre‐exudate but increased to 16.03 ± 11.86 nmol GlcNAc released cm^−2^ RSA in the post‐exudate (*p* = .199). However, it was still 5.2 times lower than that of chitosan treatment at the same concentration (Figure 4; Table S3).

Overall, the results of enzymatic assays suggest that chitosan treatment promoted defense enzyme responses and had much stronger effect than chitin treatment.

### 3.4 Chitosan induces root secretion of defense proteins into exudate

**FIGURE 5:**
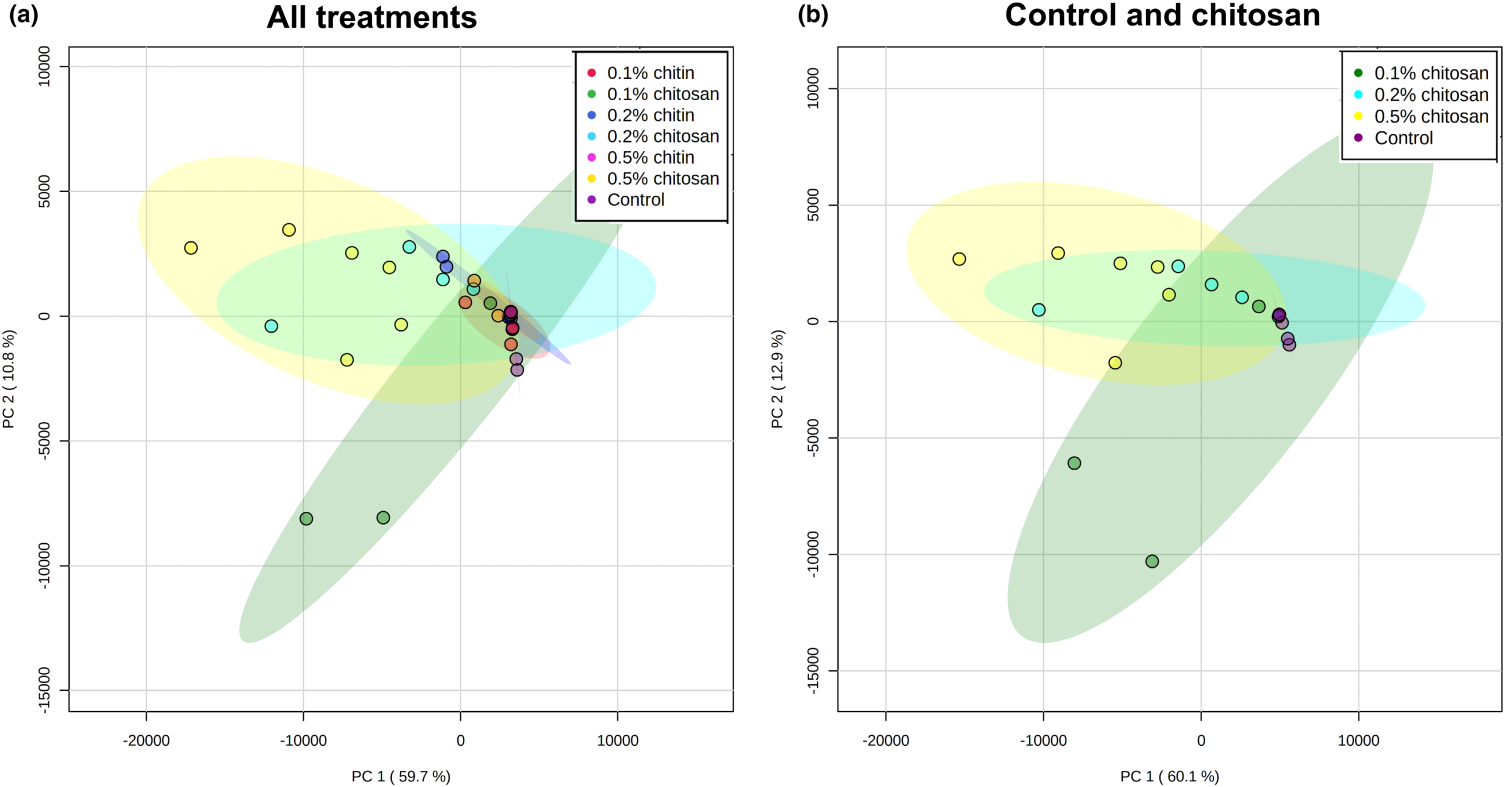
PCA plots of post‐exudate proteomes across all control, chitin and chitosan treatments (a) and selectively between control and chitosan treatments (b). Six biological replicates were analyzed per treatment. Control and chitin samples were clustered close together but two replicates of 0.1% chitosan and 0.2% chitosan and six replicates of 0.5% chitosan were clearly separated from the major assembly. The colored ellipses around each sample group represent a 95% confidence interval, drawn by MetaboAnalyst software.

**FIGURE 6:**
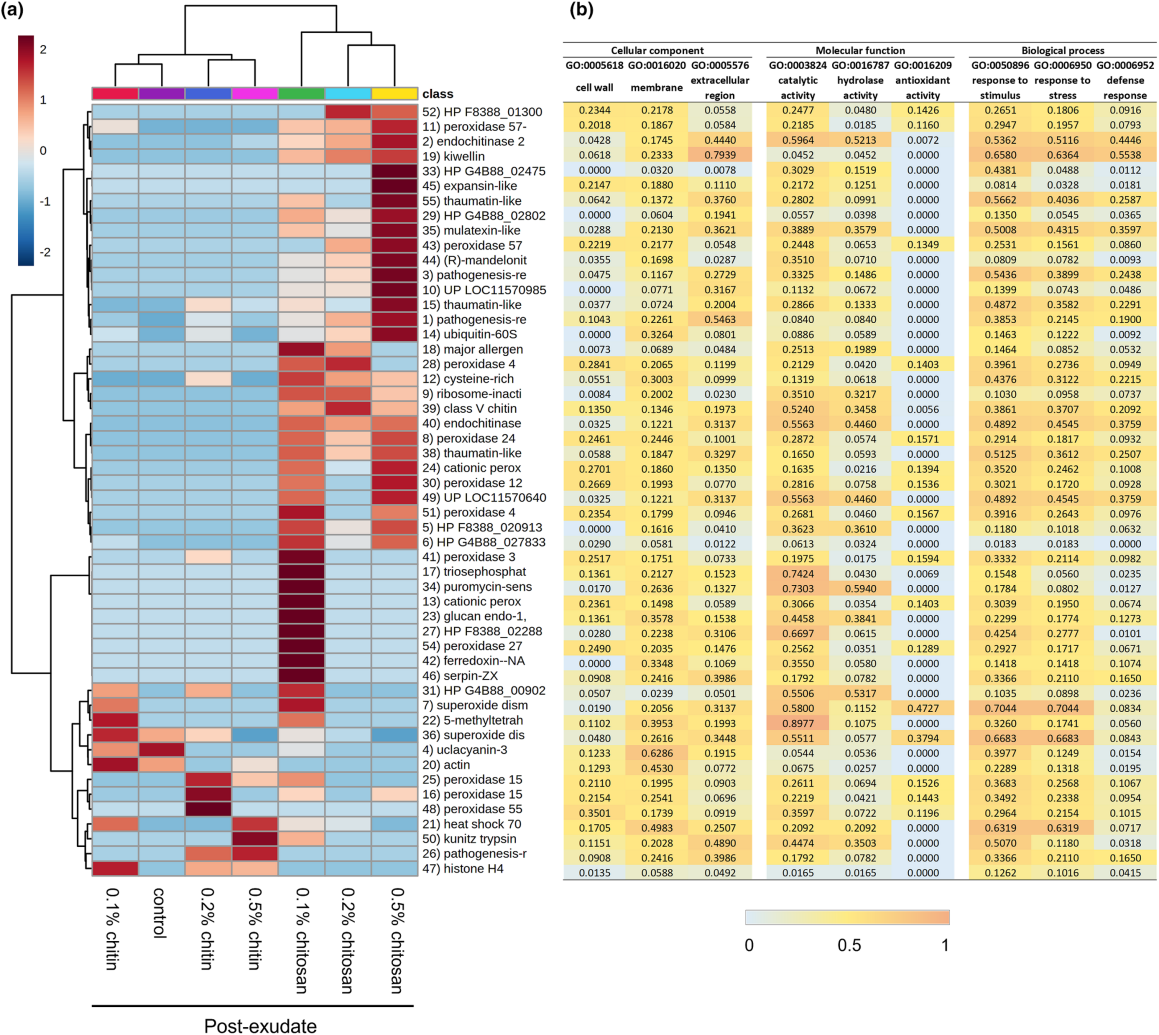
Proteomics analysis displaying clustering heatmap (a) and prediction scores (b) of the exudate proteins. Heatmap shows an average of log transformed LFQ intensity within six biological replicates per treatment (a). On horizontal axis, control and chitin samples were clustered together while chitosan treatments were grouped on a separate branch. On vertical axis, proteins highly abundant in 0.2%–0.5% chitosan treatments were clustered on the top half of the heatmap. Proteins abundant in 0.1% chitosan were branched in the middle and proteins abundant in control and chitin treatments were clustered at the bottom. Protein numbers are correlated to protein identification details listed in Table S1. Relative prediction scores are presented in three main categories of cellular location, molecular function and biological process (b). From zero to one, higher scores demonstrate an increased possibility of the protein to associate with that particular location, function and process.

Proteomic analysis on root exudate was performed to detect proteins that would be secreted into *C. sativa* root surroundings in response to chitin and chitosan treatment. To the best of our knowledge, this is the first report examining the eliciting effects of chitin and chitosan on plant exudate proteins. Across all samples, 57 protein groups were identified from the root exudates. They were assigned confidently as the identification criteria were restricted to ≥2 unique and razor peptides and ≥8% sequence coverage. Details of protein identification are found in Table S1. PCA was performed to characterize the overall difference of exudate proteomes across all samples. The plot from all samples showed that most pre‐exudates (except a small number of outliers: two replicates of 0.1% chitosan, one replicate of 0.1% chitin and one replicate of control) were clustered together, indicating close similarity in their proteome profiles before the treatments (Figure S3a). In post‐exudates, all chitin samples were aligned close to the control (Figure 5a). For chitosan treatment groups, two of the six replicates of 0.1% and 0.2% chitosan treatments were scattered away from the main cluster of control and chitin samples (Figure 5a,b). All six replicates of 0.5% chitosan were grouped together and clearly segregated from the main cluster, showing clear differences in their proteome profiles after the treatment (Figure 5b; Figure S3b). The PCA results highlight that exudate proteome profiles were changed according to chitosan treatments, whereby the clearest difference was observed in 0.5% chitosan treatment. To identify the proteins changing upon treatment, hierarchical clustering was applied and a heatmap was generated for post‐exudate samples (Figure 6a). Two major clusters were identified on the horizontal axis, where chitin treatments and control were grouped together, and chitosan treatments were grouped separately. The clustering on the vertical axis, based on relative protein response, displayed three major clusters. The proteins affected by higher concentrations of chitosan (0.2%–0.5%) were clustered on the top half of the heatmap. The proteins specifically found in 0.1% chitosan were clustered in the middle, while the proteins relative to control and chitin samples were grouped on a separate branch at the bottom (Figure 6a). The heatmap results highlight that most of the exudate proteins (approximately 40 proteins of total 57 identified proteins) were increasingly secreted upon 0.1%–0.5% chitosan treatments.

Protein function prediction was carried out based on full protein sequence using the DeepGoWeb online webserver. The results are presented in Figure 6b and Table S4 with relative score ranging from zero to one. Higher scores indicate an increased probability of the protein to associate with particular subcellular location, molecular function or biological process. Overall, several extracellular proteins such as PR protein 1, endochitinase 2, mulatexin‐like and kiwellin, were identified from the dataset. Their prediction scores were relatively high in the category of extracellular region but low in intracellular categories of cytoplasm, organelle and nucleus (Table S4). Peroxidase proteins predicted with high scores in the categories of cell wall, membrane and cell periphery were also detected in the dataset as abundant. This is reasonable because the plant constantly sloughs root end cap cells, leading to a diffusion of cell wall and membrane proteins into the exudate (Badri & Vivanco, 2009; Dubrovskaya et al., 2017). Only a few organelle or nucleolar proteins were detected. This included histone H4, ubiquitin‐60S ribosomal protein L40 and heat shock 70 kDa protein‐like (Table S4). These intracellular proteins were also identified from the root cap secretome of pea (Wen et al., 2007), suggesting they may not be uncommon proteins in extracellular region. Intracellular proteins might be derived from sloughed or broken cells and dispersed into root exudate. Overall, the result indicates the effectiveness of our sample preparation protocol and proteomics analysis to isolate and identify root exudate proteins.

Based on protein functions, half of the detected proteins are enzymes such as chitinase, peroxidase, superoxide dismutase and glucosidase (Figure 6b; Table S4). Interestingly, chitinase enzymes including endochitinase 2 and class V chitinase were identified only from chitosan treatments and classified on the top half of the heatmap (Figure 6a,b). Conversely, superoxide dismutase enzymes were found associated with control and chitin treatments, clustered at the bottom of the heatmap (Figure 6a,b). Among a variety of peroxidase isoforms, peroxidase 4, peroxidase 57 and peroxidase 24 were found in response to chitosan treatments. Peroxidase 15 and peroxidase 55 were likely to be associated with chitin treatments, but surprisingly none of peroxidase were detected from control (Figure 6a,b). In terms of biological process (Figure 6b), most of the enzymes had high prediction scores in the categories of stimulus and stress responses (>0.3000). For example, two isoforms of endochitinase 2 were predicted with 0.5362 and 0.4892 scores in the category of response to stimulus. The scores were 0.3332 and 0.7044 for peroxidase 3 and superoxide dismutase [Cu–Zn], respectively. However, only chitinase enzymes had relatively high scores in the defense response category, where two isoforms of endochitinase 2 were predicted with 0.4446 and 0.3759 scores. The defense response' scores of peroxidase 3 and superoxide dismutase [Cu–Zn] were lower than endochitinase 2, which were only 0.0982 and 0.0834, respectively. Since chitinases were increasingly secreted upon chitosan treatments, the results indicate that chitosan has potential to induce root secretion of defense enzymes into exudate. Additionally, non‐enzymatic proteins with relatively high prediction score in the category of defense response, for example PR protein R major form‐like (0.2438), thaumatin‐like protein 1b (0.2587), mulatexin‐like (0.3597) and kiwellin (0.5538) were also predominantly detected from chitosan treatments (Figure 6a,b). This emphasizes the effect of chitosan to promote defense protein and enzyme secretions.

**FIGURE 7:**
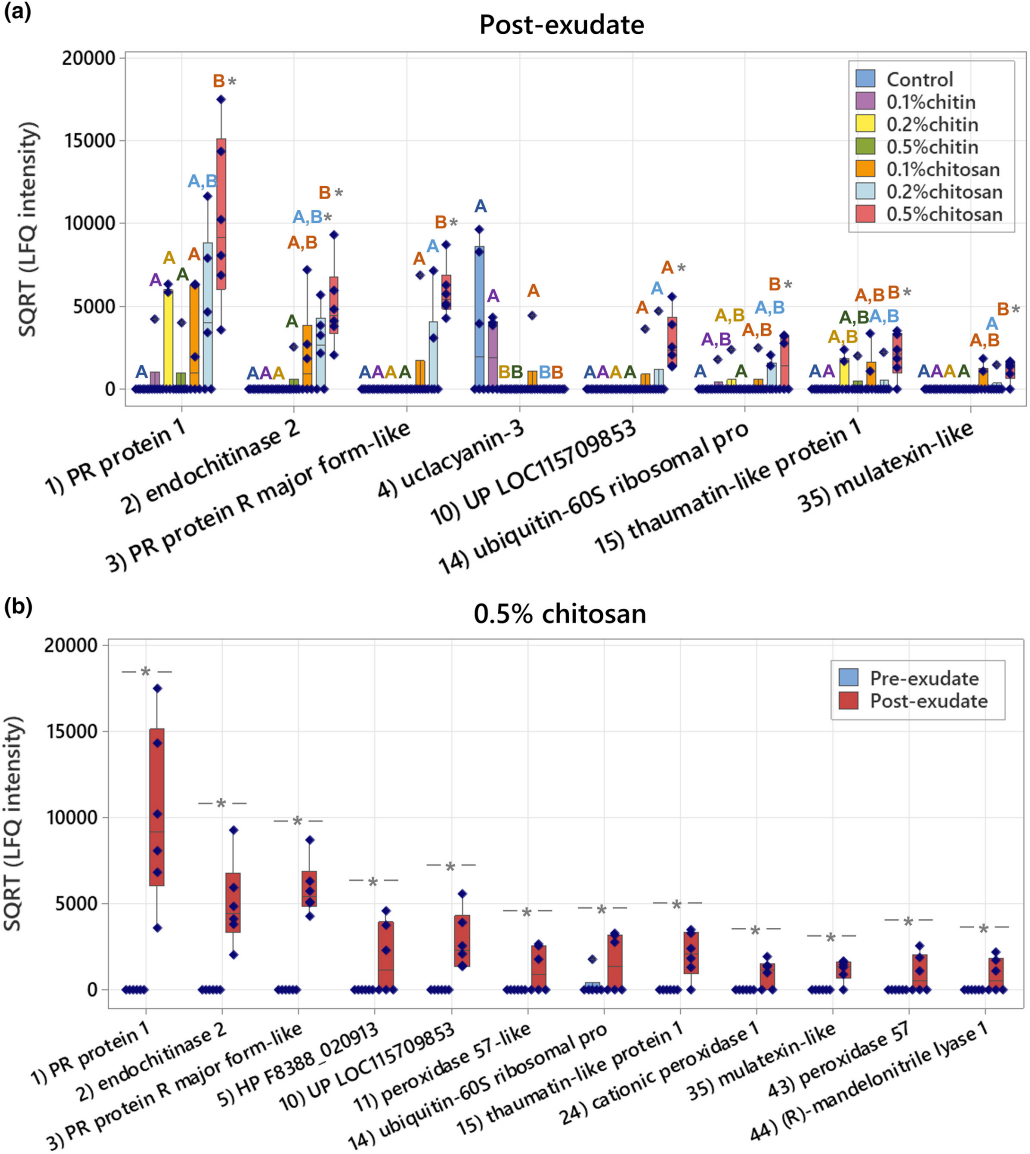
Significant proteins identified from the root exudate proteome data. (a) Eight significant proteins in the post‐exudates across all sample groups and (b) twelve significant proteins between pre‐ and post‐exudates of 0.5% chitosan treatment. The plots display interquartile range box with whiskers and individual values within six biological replicates. Letters (A and B) show significant difference (*p* < .05) across all sample groups using ANOVA test, followed by Tukey's post hoc analysis. Asterisk (*) indicates significant difference (*p* < .05) between pre‐ and post‐exudates using paired *t*‐test.

Statistical analysis was then applied to identify proteins of different levels in the exudates. Two analytical aspects were performed: (1) testing across all conditions within pre‐ or post‐exudates using one‐way ANOVA and (2) comparing between pre‐ and post‐exudates of each condition using paired *t*‐test. In pre‐exudates, there were no significantly different proteins among all studied groups (Table S1), suggesting the exudate proteomes of all samples were similar before the treatment. In post‐exudates, there were eight significant proteins detected, with seven proteins found to be highly secreted in 0.5% chitosan condition and one protein, uclacyanin‐3 (*q*‐value = .0240), found to be significantly higher in control (Figure 7a; Table S1). Comparing between pre‐ and post‐exudates, 12 proteins were significantly increased according to 0.5% chitosan treatment and one protein, endochitinase 2, was significantly higher in 0.2% chitosan condition (Figure 7b; Table S1). Interestingly, most of the significant proteins are well‐known PR proteins, such as PR protein 1, PR protein R major form‐like, endochitinase 2, thaumatin‐like protein 1 and mulatexin‐like protein, which generally function once plant experiences pest and pathogen attack (Agrios, 2005; Ferreira et al., 2007). However, none were detected as a significant protein in control, 0.1%–0.5% chitin and 0.1% chitosan treatments. This data highlights that the root exudate proteome was significantly changed upon chitosan treatments, but this phenomenon was not observed in chitin treatments.

### 3.5 Defense genes are upregulated in root tissues of chitosan‐treated plants

Based on exudate proteomics results, four significant defense‐related proteins including PR protein 1, endochitinase 2, PR‐R major form‐like and thaumatin‐like protein 1, were selected for qPCR analysis to verify exudate proteome data. Details of the selected genes are described in Table S2. Their transcript levels were quantified in the root tissues, collected on the last day of observation. Cucumber peeling cupredoxin was used as a reference gene since it was mostly detected across all the exudate samples (Tables S1 and S2). It is a copper protein, found in the cell membrane and typically plays a role in electron transport and energy production (Zhang et al., 2021).

**FIGURE 8:**
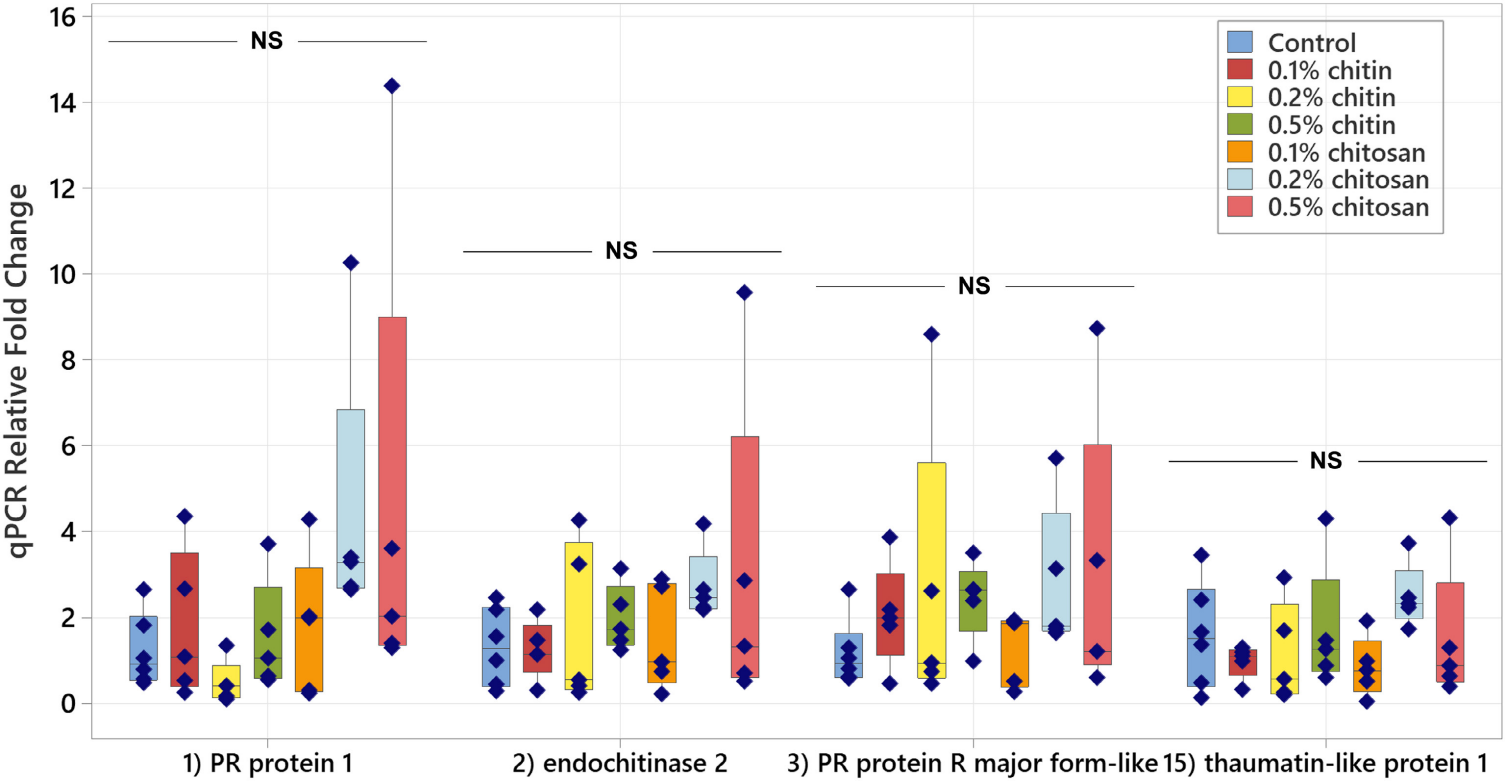
Relative transcript levels of four defense‐related genes, encoding PR protein 1, endochitinase 2, PR protein R major form‐like and thaumatin‐like protein 1, measured from root tissues of chitin and chitosan treatments as normalized to control within five biological replicates. Statistic was tested across all sample groups using ANOVA, followed by Tukey's post hoc analysis but no significant difference (NS) at *p* < .05 was found in any transcript.

Overall, transcript levels of defense genes tended to increase in root tissues of chitosan‐treated samples, although the increases were not significantly different from control and chitin treatments. Relative transcript levels of PR protein 1 were approximately 4.5‐times higher than control in the root samples of 0.2% and 0.5% chitosan treatments, with *p* = .418 and .389, respectively but only 0.48–1.77 times in other treatments (Figure 8). Likewise, transcript levels of endochitinase 2 were 2.73 and 2.99 times higher than control in 0.2% and 0.5% chitosan samples, with *p* = .825 and .693, respectively but only 1.24–1.97 times different in other treatments. The levels of PR protein R major form‐like gene were more than two‐times higher than control in 0.2% and 0.5% of both chitin and chitosan treatments (*p* = .829 and .738, respectively). The level of thaumatin‐like protein 1 was 2.48‐times higher than control in 0.2% chitosan treatment (*p* = .832). Insignificant increases of transcript levels in the root tissues of chitosan treatment could be explained by a couple of reasons. For example, the increasing levels of PR protein 1, endochitinase 2 and PR protein R‐major form‐like were detected with high variation within chitosan sample groups, suggesting plants might respond to chitosan to a different degree, and more sample replicates would be required for further analysis to draw a conclusive outcome. Sampling timepoint could be another factor, where gene expression processes could take place at the early stage once the plant was initially exposed to chitosan and decline over time (De Vega et al., 2021; Lopez‐Moya et al., 2017). In addition, the production of a single protein could depend on multiple genes, and a myriad of other genes could be involved in the protein secretion process (Kwon et al., 2008). Therefore, significant increase of the proteins detected from the exudate may not be attributed to a single gene or transcript in the root tissues. Further investigation is required to closely examine the effect of chitosan on the regulations of defense‐related genes in the root tissues, where the analysis should be performed in a time series manner.

Despite statistically insignificant differences, the inducing effect of chitosan on PR protein 1, endochitinase 2 and PR protein R major form‐like genes were likely to be dose dependent since positive linear relationships were observed from the plots between qPCR relative fold changes and chitosan concentrations (Figure S4). Furthermore, this qPCR transcript data was linked with the exudate proteomics and bioassay results to find a correlation between different technical analyses. After removing one outlier sample of 0.5% chitosan treatment, a positive correlation between transcript level and exudate protein intensity was observed from endochitinase 2 in 0.2% and 0.5% chitosan treatments (Figure S5). The correlations were also positive for the other proteins, including PR protein 1, PR‐R major form‐like and thaumatin‐like protein 1, within 0.5% chitosan treatment. However, the correlation was negative in the lowest chitosan concentration (0.1%) of all proteins (Figure S5). This implies that the increased regulation of defense genes in the root tissues likely yield the increased secretion of the proteins in the exudates, but there could be other biological factors involving during protein production and secretion processes. Additionally, endochitinase 2 transcript level was found to be negatively correlated with total chitinase activities measured from root tissues and exudates (Figure S6), suggesting that total chitinase activities in the tissue and exudate were not influenced by a single chitinase protein but potentially co‐contributed by other chitinases.

## 4 DISCUSSION

This study aimed to investigate the effects of chitin and chitosan to promote overall defense responses of the *C. sativa* root system. The results demonstrate that chitosan has a much stronger effect than chitin to activate plant root defense responses. Chitosan can induce the production of defense enzymes in root tissue (Figure 4) and the secretion of defense proteins into root exudate (Figures 6 and 7). The gene transcript levels of defense proteins were also upregulated (Figure 8), and cellular levels of ABA and CA were increased in root tissues (Figure 3), but the changes of defense hormone levels including SA and JA were not clearly detected. In contrast, none of the defense responses induced by chitosan were observed in chitin treatments.

In general, chitin and chitosan may be considered relatively similar in structure. Both are natural β‐1,4‐linked polysaccharides, with the predominant subunit of *N*‐acetyl‐D‐glucosamine and D‐glucosamine for chitin and chitosan, respectively. The difference is the absence of the acetyl group in chitosan subunits. Chitin may be transformed to chitosan by natural chitin deacetylase enzymes (Zhao et al., 2010) or chemical deacetylation reactions (Vicente et al., 2021). However, the conversion may not be fully complete, and chitosan may be present in a partially acetylated form. The degree of deacetylation determines the number of acetyl groups removed from the chitosan structure. This value along with crystallinity, viscosity and polymer size affect the chemical and physical properties of chitosan (Triunfo et al., 2022).

In plants, the outer cell membrane has receptors which can specifically bind to chitin. Chitin‐binding receptor is a protein complex, containing multiple lysin motif (LysM) domain proteins and receptor‐like kinases (RLKs) or receptor‐like proteins (RLPs) (Shinya et al., 2015). Chitin oligomers (or short‐chain chitins) have high affinity to bind the receptor (Cao et al., 2014; Gubaeva et al., 2018; Hayafune et al., 2014). Interactions between chitin oligomers and chitin‐binding receptors and downstream signaling processes may vary across different plant species but ultimately binding results in an activation of plant cellular defense response (García et al., 2021). The signaling process is rendered through mitogen‐activated protein kinase (MAPK) cascades and in association with plant defense hormones, i.e., salicylic acid, jasmonic acid and ethylene (Gong et al., 2020; Ramirez‐Prado et al., 2018). On the other hand, plant receptors that can specifically bind to chitosan have not yet been reported (Yin et al., 2016), even though chitosan has been well described as a plant elicitor to induce plant defense (Pichyangkura & Chadchawan, 2015). The mechanism underlying chitosan‐promoted plant defense is still unclear. It could be via chitin‐binding receptors because partially deacetylated chitosan retains chitin‐like identity and may be able to bind to the receptors. Chitosan with 60% deacetylation had higher eliciting activity than chitin oligomer (8 subunits) to enhance H_2_O_2_ production in Arabidopsis seedlings (Gubaeva et al., 2018). However, fully deacetylated chitosan had no effect, suggesting the acetyl group in chitin or partially deacetylated chitosan is required for chito‐polymers to interact with plant receptors (Gubaeva et al., 2018). The chitosan used in our study was 75%–85% deacetylated (Sigma‐Aldrich, 2022). Hence, chitosan‐induced defense responses as observed might be triggered via the binding of chitosan to chitin‐binding receptors. Nevertheless, it is also feasible that the effects were induced via the binding of chitosan to its own specific receptors, which have not yet been discovered. Alternatively, chitosan may alter plant cell membrane permeability and directly signal cytoplasmic secondary messengers, such as hydrogen peroxide, nitric oxide, and phytohormones, triggering their downstream pathways (Pichyangkura & Chadchawan, 2015), or bind to chromatin in the nucleus to regulate plant defense responses (Hadwiger, 2013).

Our results also suggest that exogenous intact chitin poorly activates plant defense responses, likely due to inability to bind plant cell membrane receptors (Shinya et al., 2015). Based on scanning electron microscope, crude chitin aggregates into particles approximately 20 μm in diameter which is much larger than approximate size, 8 nm in diameter, of the extracellular domain of toll‐like receptor, one of the classical receptors in plant and animal immune system (Fuchs et al., 2018). This large size mismatch and lack of available soluble chitin may impede direct binding between crude chitin and eukaryotic cell membrane receptors. Long‐chain chitin nanofibers (chitin fibrils embedded in a protein matrix) can enhance cellular H_2_O_2_ production and PR gene expressions in Arabidopsis, but it is modified to be highly dispersed in water and loosely agglomerated, thereby can be easily degraded by chitinase enzymes (Egusa et al., 2015). Our results support the concept that natural interaction between fungal chitin and plant receptors requires digestion steps to break down polymeric cell wall chitin into soluble, recognizable chitin fragments (Shinya et al., 2015).

**FIGURE 9:**
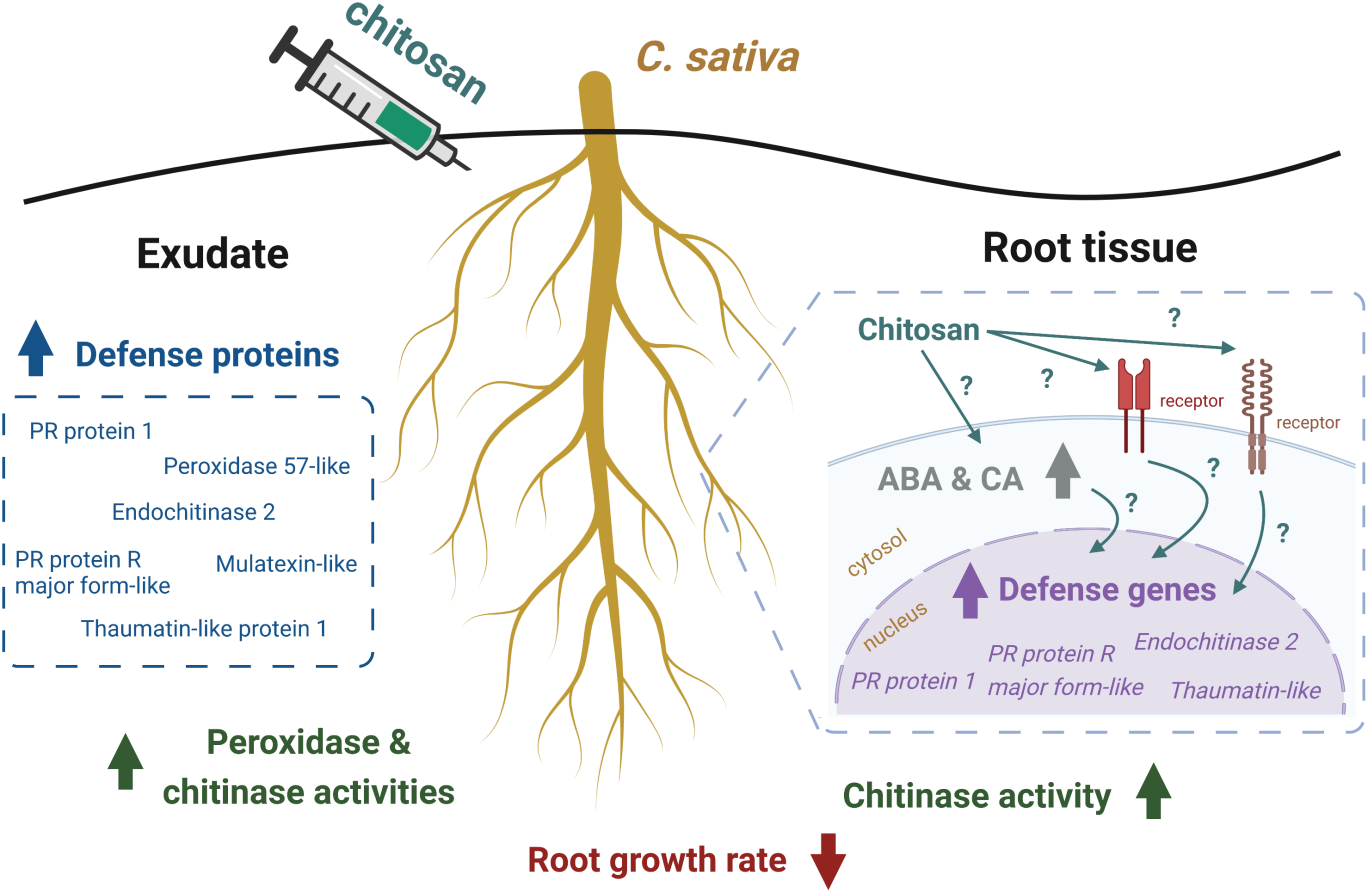
Summary of *Cannabis sativa* root responses upon colloidal chitosan treatment as observed in this study. Root growth was affected immediately after the treatment. In the root tissue, levels of ABA hormone and CA compound were increased, defense genes were upregulated and total chitinase activity was enhanced. In the exudate, increased secretion of several defense proteins such as PR protein 1 and endochitinase 2 was detected and total peroxidase and chitinase activities were higher than control and pre‐treatment. Biological mechanisms underlying root responses caused by chitosan remain uncharacterized. This figure was created with Biorender.com.

On the other hand, enhanced defense responses as observed from chitosan treatments have confirmed its eliciting properties (Chandra, Chakraborty, et al., 2017; Lopez‐Moya et al., 2021; López‐Velázquez et al., 2021). As summarized in Figure 9, our analyses using various techniques (including enzymatic assays, exudate proteomics and qPCR method) were well correlated, showing increased peroxidase and chitinase activities, transcript levels of defense genes in the root tissues and increased secretion of defense proteins in the exudates. For peroxidase and chitinase activities, the increases were significantly detected in the higher concentrations (0.2% and 0.5%) of chitosan treatments. It was also observed in the 0.1% chitosan treatment but to a lower extent, suggesting that the eliciting properties of chitosan on plant defense enzymes could be dose dependent. However, enzymatic activities of chitin treatments were not significantly changed in any concentrations, reiterating an inability of intact chitin to enhance defense enzyme responses (Figure 4). Nonetheless, due to high intra‐variation observed within sample groups of chitin and chitosan treatments, additional observation, potentially examining at an earlier timepoint and/or younger plant stage, is required to confirm the effects of chitin and chitosan on plant defense enzymes. It is also worthwhile to expand the examination to other enzymes, for instance catalase, superoxide dismutase and chitosanase.

In the root exudate proteome data, the detection of multiple defense proteins, for example PR protein 1, PR protein R major‐form‐like and thaumatin‐like protein 1 in the exudates of chitosan treatments emphasized the eliciting effect of chitosan on plant root systems (Figures 6 and 7). The effect also appeared to be dose dependent as it was strongest in the 0.5% chitosan treatment and gradually lower in the 0.2% and 0.1% chitosan treatments. To the best of our knowledge, this is the first report showing increased secretion of defense proteins into exudate in response to chitosan. The majority of significantly increased proteins in exudate are classified as PR proteins which are usually increasingly expressed once a plant is attacked by pathogens. PR protein 1 has long been used as a genetic marker for plant systemic acquired resistance (Breen et al., 2017). Chitinases are key enzymes that protect plants against fungal pathogens (Kaur et al., 2022; Vaghefi et al., 2013). Thaumatin‐like protein has been shown to play a role in wheat resistance against leaf rust fungus, *Puccinia triticina* (Zhang et al., 2018). Mulatexin protein has a strong toxicity against silkworms, helping protect mulberry tree from pest attack (Wasano et al., 2009). Additionally, a previous study on tomato root demonstrated that chitosan could depolarize root cell membrane and induced secretions of phytohormones, lipid signaling and phenolic compounds in root exudate (Suarez‐Fernandez et al., 2020). Combining this information with our root exudate proteome data, it could be concluded that the impact of chitosan is not limited to plant tissues but can be extended to root metabolite and protein secretions.

The molecular mechanism underlying chitosan promotion of plant defense is still unclear. We hypothesized that chitosan could mediate biosynthesis of phytohormones. In this study, phytohormone measurement showed significant increases of ABA hormone and CA compound in the root tissue of 0.5% chitosan treatment. The levels of known defense hormones, SA and JA, were not significantly changed in any conditions, but SA, JA, JA‐Ile and OPDA levels were slightly increased in 0.2% and 0.5% chitosan treatments (Figure 3). Chitosan might alter these defense hormone responses, but the sampling timepoint could be a factor influencing phytohormone data in our experiment. The tissue samples were collected one week after the treatment, but upregulations of genes or transcription factors related to phytohormone biosynthesis could happen within 24 h after chitosan treatment (Colman et al., 2019). The higher level of ABA detected suggests that ABA might contribute to the increased levels of defense genes and proteins. ABA has long been proposed to associate with abiotic stress, but recently its role in relation to biotic stress has been increasingly reported (Shigenaga & Argueso, 2016). ABA signaling pathway cross talks with SA and JA defense hormone pathways, implying ABA is also involved in regulating plant disease resistance (Anderson et al., 2004; Berens et al., 2017). This is evidenced by several studies, for example, spraying 100 μM of exogenous ABA induced expression levels of PR4 and PR10 genes in lentil seedlings (Ford et al., 2017). Additionally, ABA hormone can modulate rice MAPK5 gene and protein biosynthesis, influencing downstream signaling processes of both disease resistance and abiotic stress tolerance (Xiong & Yang, 2003). Therefore, ABA could be one of the players, triggering defense responses in *C. sativa* root system as observed.

Moreover, we found that root growth was largely interrupted by chitosan, which is consistent with previous observations in Arabidopsis root (Iglesias et al., 2019; Lopez‐Moya et al., 2017). The inhibition is supposedly due to upregulation of transcription factors involved in the auxin biosynthesis pathway, resulting in auxin accumulation at the root tips (Lopez‐Moya et al., 2019). In our study, auxin levels, IAA and Me‐IAA, were comparable to control in the root tissues but slightly decreased in the shoots. Based on these results, chitosan may disrupt the balance of auxin levels in plant shoot and root tissues, affecting both shoot and root growth, but this interpretation needs further investigation to warrant the fact. It is also possible that auxin accumulation at the root tips may take place at the early stage after chitosan exposure. The effect may ease off through time as expression level of the *YUC2* gene, encoding one of the key enzymes in the IAA biosynthesis pathway, leveled off within three days after the treatment (Lopez‐Moya et al., 2017). Increases in cellular CA level may be another factor affecting root growth since treating plants with exogenous CA were evidenced to decrease root length, alter activity of IAA oxidase, an IAA degradation enzyme and inhibit auxin efflux (Salvador et al., 2013; Steenackers et al., 2017). In addition, defense hormones, SA, JA and its derivatives, could be another factor driving the changes in root development, since defense hormones are known to crosstalk with growth hormones to balance overall plant activities (Berens et al., 2017; Denancé et al., 2013) and their levels in this study were slightly increased along the increasing concentrations of chitosan treatments, where root growth was interrupted. Additional investigation is required to delve into the interactions between defense hormone activation and root growth disruption, caused by chitosan.

Ultimately, the stalling of root growth suggests that the plant may transform energy from expanding roots to consolidate defense system in response to chitosan. This plant adaptation process is known as growth‐defense tradeoff, which can be triggered by any abiotic and biotic factors (He et al., 2022; Huot et al., 2014). Our findings demonstrate that chitosan appears to activate this process in *C. sativa* root system. Further exploration is required to identify the biological mechanisms underlying this process upon chitosan treatment. The knowledge gained would benefit crop improvement to maximize crop yield with a balance of disease resistance (Silva et al., 2019). After all, chitosan could be a potential elicitor for agricultural application, especially in hydroponic setup to counteract fungal pathogen attacks under safe and practical conditions.

## 5 CONCLUSION

Finding safe and effective solutions to manage crop diseases is an essential task to support *C. sativa* production. Chitin and chitosan are natural elicitors, known to promote plant defense. They may be similar in terms of structure, but their effects on plant cell recognition, physiological responses and molecular processes are highly distinct. We found that colloidal chitin has very low impact on *C. sativa* defense promotion while colloidal chitosan can enhance defense responses in root tissue and exudate. The key finding was the detection of several defense proteins including PR proteins, chitinases and thaumatin‐like proteins that were increasingly secreted upon chitosan treatment. This was confirmed by increases in total activities of peroxidase and chitinase enzymes in the exudate. However, root growth was interrupted after chitosan exposure. Biological pathways underlying defense promotion, but root growth inhibition caused by chitosan remain uncharacterized. Increased cellular levels of ABA and CA were detected and could be one of the underlying factors. Further study is required to investigate how the plant recognizes the chitosan molecules and what signaling pathways lead to the root transformation from growth to defense. Nonetheless, chitosan has potential for implementation in *C. sativa* production, particularly in hydroponic cultivation to manage waterborne fungal diseases.

## CONFLICT OF INTEREST STATEMENT

This work was partly financially supported by Nutrifield Pty Ltd.

## Supporting information


Figure S1

Figure S2

Figure S3

Figure S4

Figure S5

Figure S6

Table S1

Table S2

Table S3

Table S4

## Data Availability

The data that support the findings of this study are available within the paper or in the supplementary materials published online.
