# Supplementary figures and images for: Effects of chitin and chitosan on root growth, biochemical defense response and exudate proteome of Cannabis sativa
Source: Plant Environ Interact. 2023 Apr 2;4(3):115–33. doi: 10.1002/pei3.10106 (PMC10290428; doi:10.1002/pei3.10106)

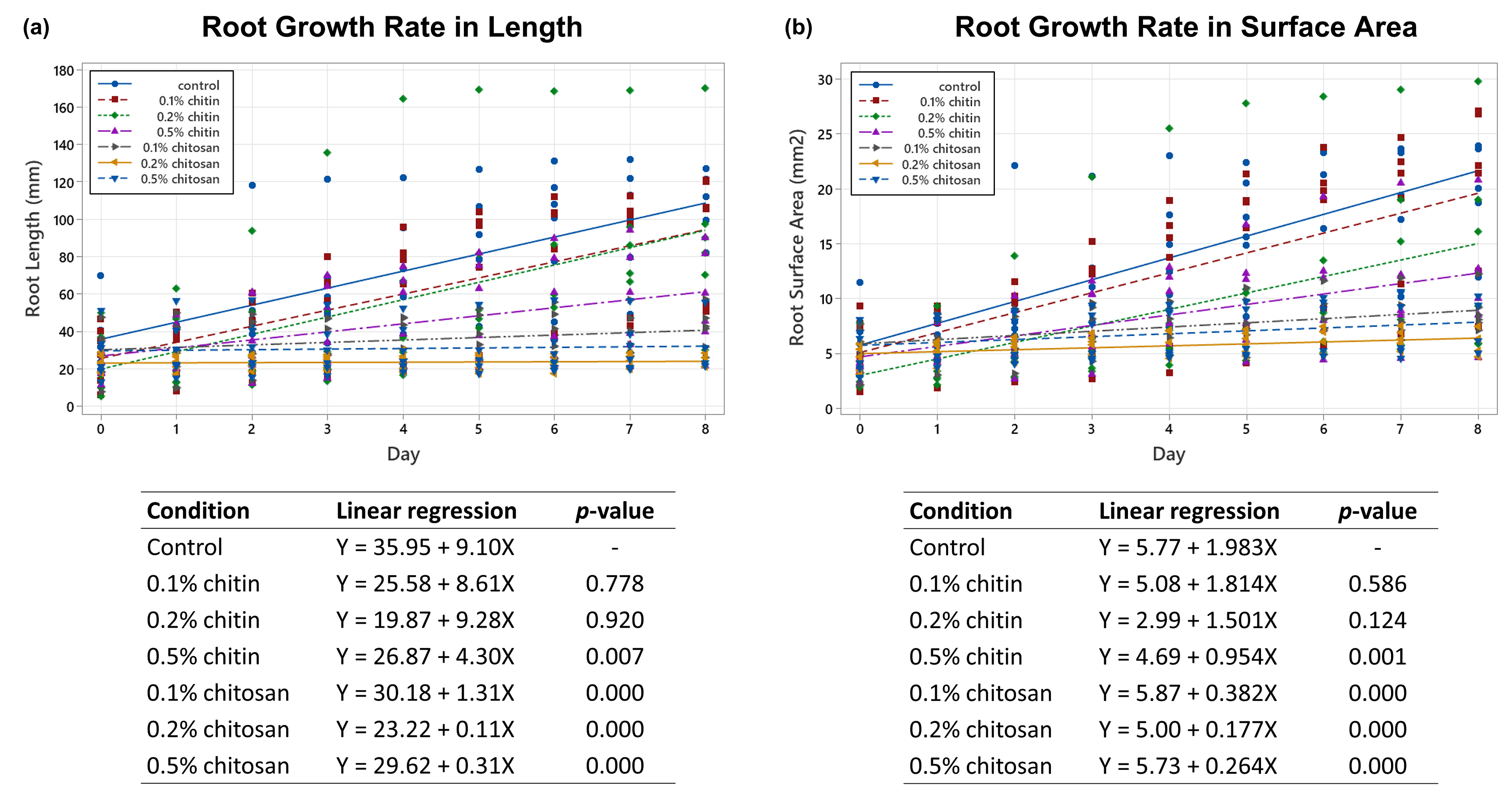

Supplement: Supplementary file 1 — Figure S1 [file PEI3-4-115-s003.tif]

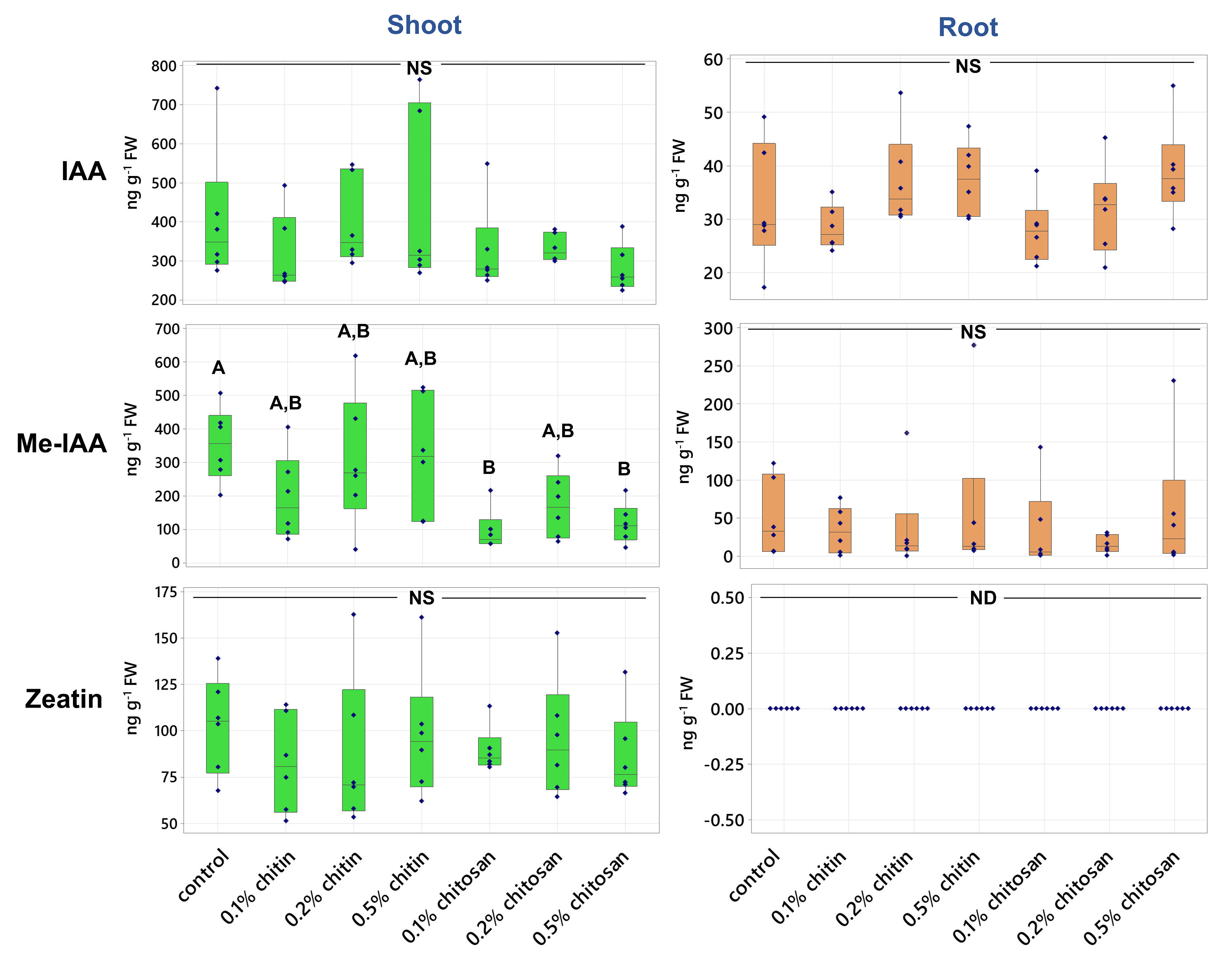

Supplement: Supplementary file 2 — Figure S2 [file PEI3-4-115-s002.tif]

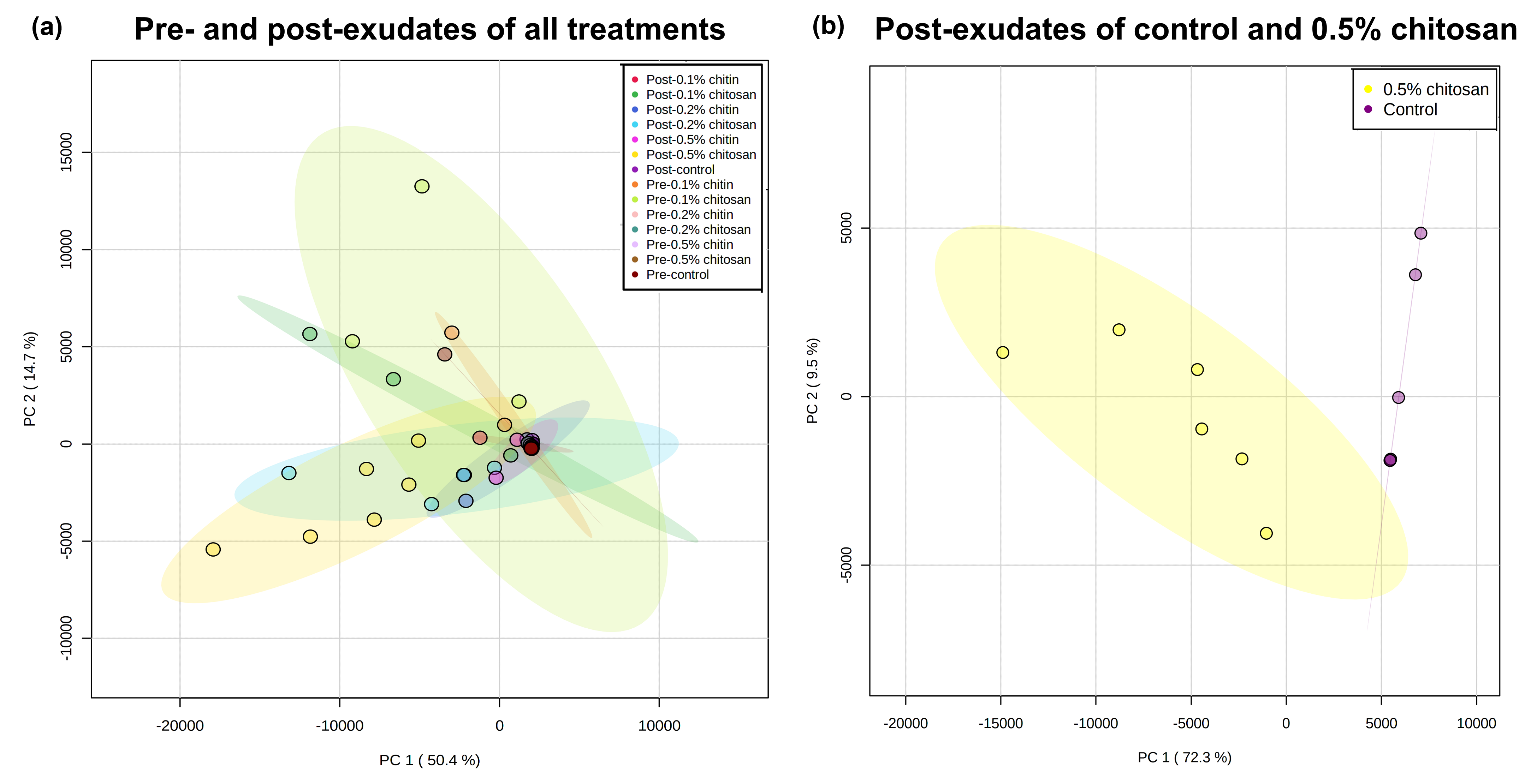

Supplement: Supplementary file 3 — Figure S3 [file PEI3-4-115-s005.tif]

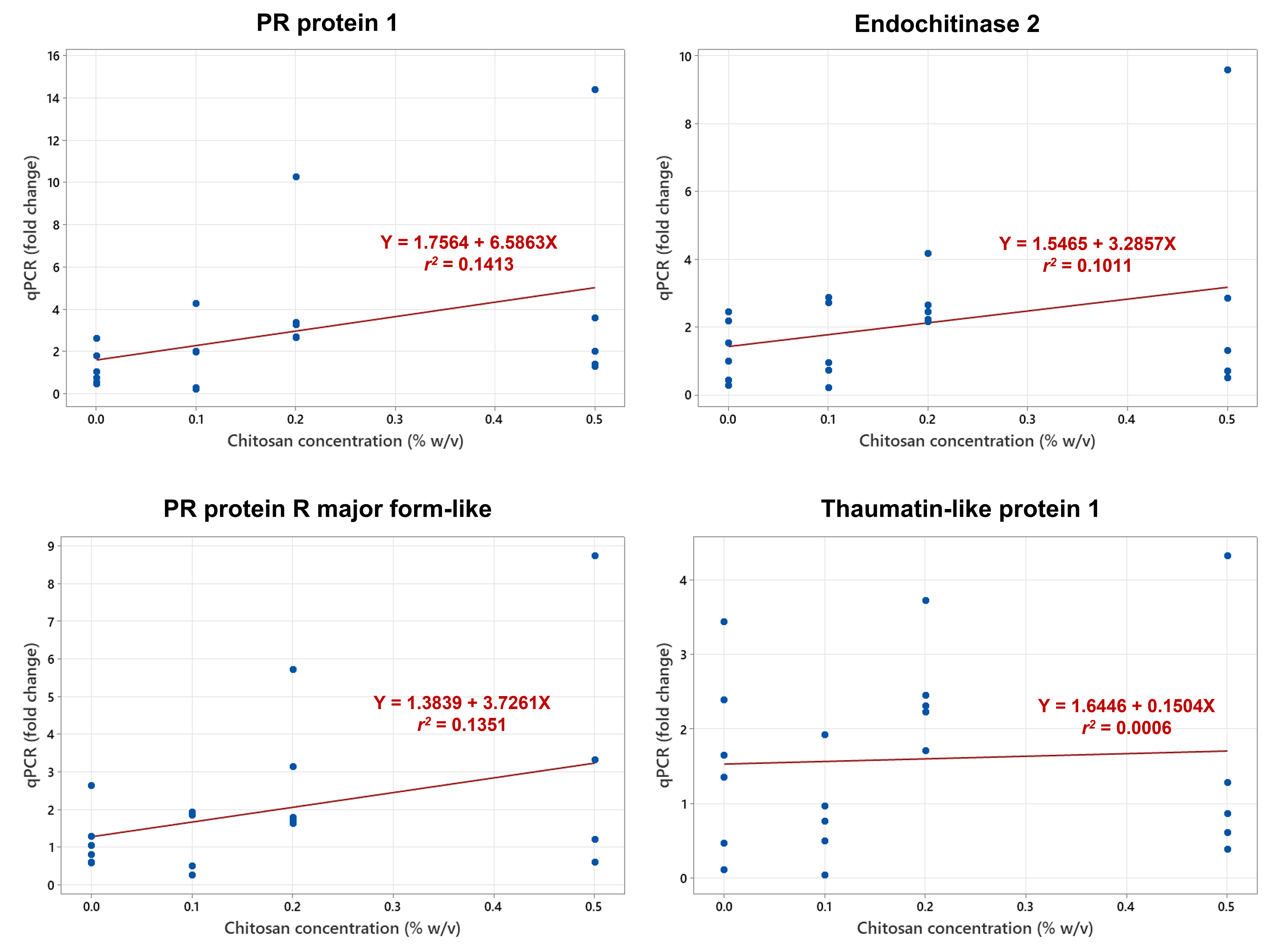

Supplement: Supplementary file 4 — Figure S4 [file PEI3-4-115-s009.tif]

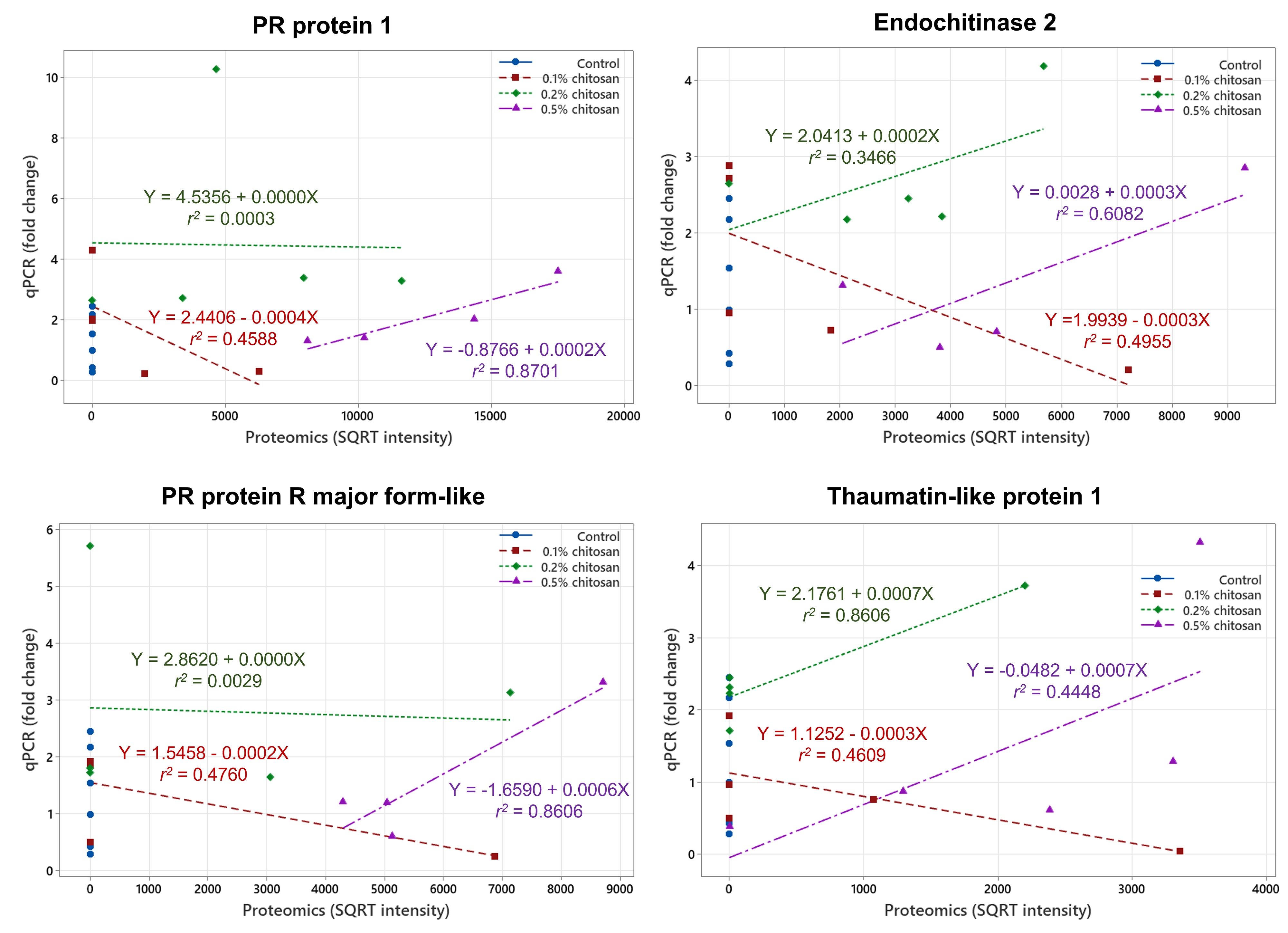

Supplement: Supplementary file 5 — Figure S5 [file PEI3-4-115-s010.tif]

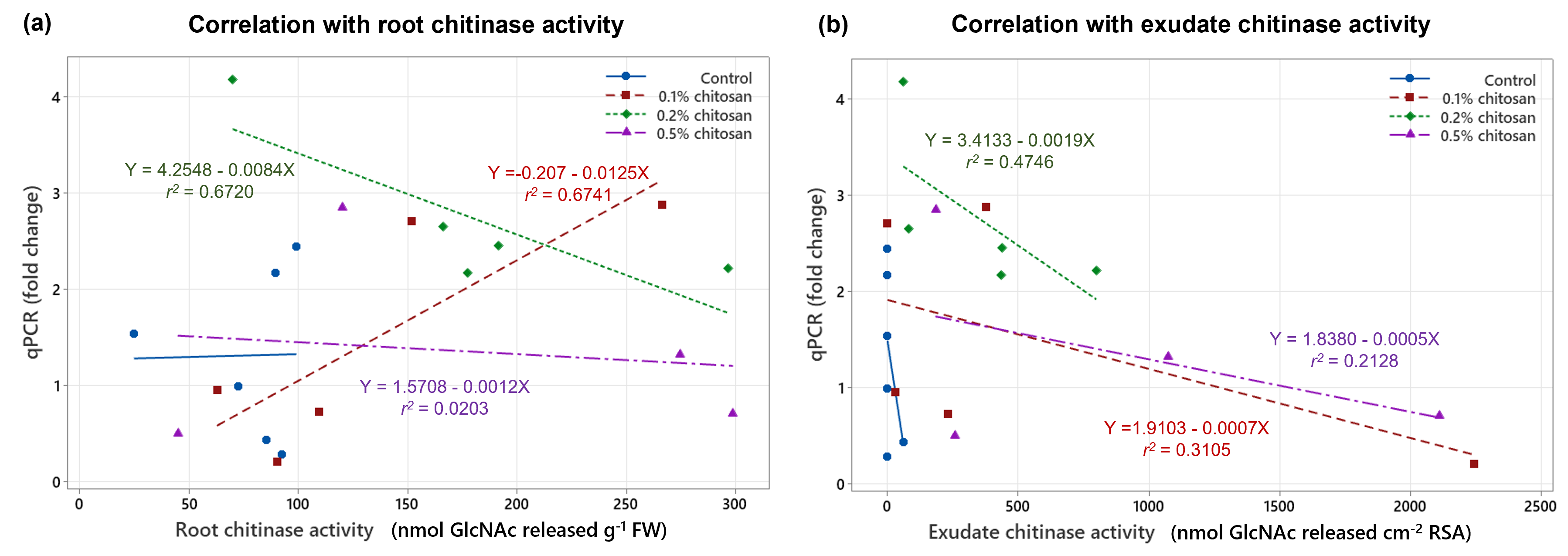

Supplement: Supplementary file 6 — Figure S6 [file PEI3-4-115-s008.tif]
